# Cellular and pathological heterogeneity of primary tauopathies

**DOI:** 10.1186/s13024-021-00476-x

**Published:** 2021-08-23

**Authors:** Dah-eun Chloe Chung, Shanu Roemer, Leonard Petrucelli, Dennis W. Dickson

**Affiliations:** 1 Department of Neuroscience, Mayo Clinic, 32224 Jacksonville, FL USA; 2 Department of Molecular and Human Genetics, Baylor College of Medicine, 77030 Houston, TX USA; 3 Jan and Dan Duncan Neurological Research Institute, Texas Children’s Hospital, 77030 Houston, TX USA

**Keywords:** Animal models, Astrocytes, Cryo-EM, MAPT, Microglia, Oligodendroglia, Tau, Tauopathy, Transcriptomics

## Abstract

Microtubule-associated protein tau is abnormally aggregated in neuronal and glial cells in a range of neurodegenerative diseases that are collectively referred to as tauopathies. Multiple studies have suggested that pathological tau species may act as a seed that promotes aggregation of endogenous tau in naïve cells and contributes to propagation of tau pathology. While they share pathological tau aggregation as a common feature, tauopathies are distinct from one another with respect to predominant tau isoforms that accumulate and the selective vulnerability of brain regions and cell types that have tau inclusions. For instance, primary tauopathies present with glial tau pathology, while it is mostly neuronal in Alzheimer’s disease (AD). Also, morphologies of tau inclusions can greatly vary even within the same cell type, suggesting distinct mechanisms or distinct tau conformers in each tauopathy. Neuropathological heterogeneity across tauopathies challenges our understanding of pathophysiology behind tau seeding and aggregation, as well as our efforts to develop effective therapeutic strategies for AD and other tauopathies. In this review, we describe diverse neuropathological features of tau inclusions in neurodegenerative tauopathies and discuss what has been learned from experimental studies with mouse models, advanced transcriptomics, and cryo-electron microscopy (cryo-EM) on the biology underlying cell type-specific tau pathology.

## Overview of tau physiology and aggregation pathobiology

Tau is a microtubule-associated protein that promotes microtubule assembly and stability. Tau is encoded by a single gene (*MAPT*) on chromosome 17q21, with at least 16 exons, some of which undergo developmentally regulated alternative spicing to generate a family of isoforms. Tau has several functionally distinct domains (numbering according to the longest isoform), including an amino (N)-terminal projection domain (1-150), a central proline-rich domain (151–243), a repeat domain (244–368), and a carboxyl (C)-terminal domain (369–441) [1]. Due to alternative splicing of exons 2, 3, and 10, tau can be expressed in six different isoforms in the CNS, with different numbers of N-terminal inserts (0 N, 1 N, or 2 N) and either three of four repeats in the repeat domain (3R or 4R). Fetal brains have only 3R tau, but adult human brains express both 3R and 4R tau in approximately 1:1 ratio [2–4]. Initially, tau was thought to be exclusively a neuronal protein due to its robust expression in neurons [5]; however, it has been shown that oligodendrocytes and astrocytes also express tau, albeit at lower levels [6, 7].

Tau is a phosphoprotein with numerous serine and threonine residues that can be differentially phosphorylated [1]. Site-specific hyperphosphorylation of tau is one of the major hallmarks of neurodegenerative tauopathies, in which neuronal and glial cells exhibit various intracellular tau inclusions [8]. Tau is also subject to other post-translational modifications (PTMs), such as acetylation, ubiquitination, and cleavage [9–11]. Site-specific PTMs of tau can substantially impact the pathophysiology of tau by altering the solubility of tau or disrupting proper interaction between tau and microtubules [8, 9].

Intrinsically, tau is highly hydrophilic and maintains its native unfolded structure; however, under pathological conditions, tau becomes abnormally misfolded and aggregated, which impairs its physiological functions. Specifically, aggregation of tau is defined by formation of tau polymers that are often filamentous with ordered structures derived from aberrant intermolecular interaction, which is speculated to occur via a nucleation-elongation mechanism [1]. After oligomeric, aggregation-competent tau forms nuclei, they can be subsequently elongated into multimeric, filamentous structures [12, 13]. Of note, the first report of paired helical filaments (PHFs) in Alzheimer’s disease (AD) brains preceded the discovery of tau [14]. These filaments were later confirmed to be predominantly composed of hyperphosphorylated tau (p-tau) [15], demonstrating that tau can pathologically aggregate in neurons to form neurofibrillary tangles (NFTs). NFTs are pathological, but not necessarily associated with cell death in that NFT-bearing neurons can survive for decades [16]. This has led to the controversial suggestion that formation of NFTs could be a protective mechanism [17]. In addition to NFTs, tau aggregates form a wide range of structures depending on the neurodegenerative tauopathy, varying in vulnerability of brain regions and cell types. Such will be the focus of this review.

Pathological tau has been suggested to act as a “seed” that recruits or promotes soluble tau into further aggregation [18]. A number of animal studies have demonstrated that tau seeds can transmit pathological tau by intracerebrally injecting recombinant tau protein [19, 20], lysates of cells that express pathological tau strains [21, 22], or brain tissue-derived tau seeds [23–28] into wild-type (WT) or tau transgenic mice. Intriguingly, human brain-derived tau seeds injected into mouse brains have been shown to recapitulate heterogeneity of neuropathological lesions of the disorders from which the tau protein was obtained, in terms of morphology of lesions, affected cell types and vulnerable brain regions [21–28]. Complementary observations were made in cellular models in which tau seeds derived from various human tauopathies demonstrated different seeding potency and induced tau aggregates with unique morphological features [21, 28, 29]. It should be noted that in most of these studies, the seeding materials are not pure tau, but contain of factors that copurify with pathologic tau. Nevertheless, these studies collectively suggest that distinct tau strains or distinct conformers may underlie neuropathological heterogeneity of tau pathology in neurodegenerative tauopathies, including cell-type specificity.

## Tauopathies

Tauopathies are a group of neurodegenerative diseases that are histopathologically characterized by abnormal accumulation and aggregation of tau within neurons or glial cells, or both. In addition to diverse clinical presentations, tau lesions in tauopathies are heterogeneous in their isoform composition, morphology, cell type involvement, and brain regions most affected (Table 1). Tauopathies are divided into primary tauopathies and secondary tauopathies. The latter is defined by substantial aggregation of a second protein, which is considered as the primary driver of the disease process. The most common example of a secondary tauopathy is AD, where tau pathology is thought to be driven or accelerated by beta-amyloid (Aβ) [30]. Of note, while many primary tauopathies are considered to be at least partially driven by genetic factors, a subset of tauopathies are more clearly linked to environmental triggers or exposure, such as repeated (sub)concussions in chronic traumatic encephalopathy (CTE) and environmental neurotoxins in Guam Parkinsonism-dementia complex (PDC) [31, 32].

**Table 1:** A list of different tauopathies and their cell type-specific tau lesions

Tauopathy		Tau lesions		
**Disorder**	**Predominant tau isoform**	**Neuron**	**Astrocyte**	**Oligodendrocyte**
PiD	3R	Pick bodies (+++) and Ballooned neurons (++)	Ramified astrocytes	Pick body-like inclusions
PSP	4R	Neurofibrillary tangles (++) and Pretangles (+++)	Tufted astrocytes	Coiled bodies(+++)
CBD	4R	Pretangles (+++) and Ballooned neurons(++)	Astrocytic plaques	Coiled bodies(++)
GGT	4R	Pretangles (+++)	Globular astrocytic inclusions	Globular oligodendroglial inclusions
AGD	4R	Grains (+++) and Ballooned neurons(+)	Ramified (bushy) astrocytes	Coiled bodies(+)
PART	3R/4R	Neurofibrillary tangles	N/A	N/A
ARTAG	4R	N/A	Thorn-shaped and Granular fuzzy astrocytes	N/A
AD	3R/4R	Neurofibrillary tangles (+++)	N/A	N/A
CTE	3R/4R	Neurofibrillary tangles (++)	Thorn-shaped astrocytes	N/A

Abbreviations: +: mild; ++: moderate; +++: marked; *N/A* not applicable

### Primary tauopathies

#### Frontotemporal dementia

Frontotemporal dementia (FTD) is an umbrella term for various clinical syndromes ranging from personality and behavioral changes to language impairment to movement disorders [33]. Neuropathologic substrates of FTD are heterogeneous, but they show anatomical overlap, with frontal and temporal lobes being selectively vulnerable to degeneration. The term frontotemporal lobar degeneration (FTLD) is used to classify the pathology and molecular underpinnings of the clinical FTD syndrome [33]. FTLD-tau is a major molecular subgroup of FTLD that is characterized by aberrant accumulation of ptau in neurons and glia. The major subtypes of FTLD-tau are Pick’s disease (PiD), corticobasal degeneration (CBD), progressive supranuclear palsy (PSP), globular glial tauopathy (GGT), frontotemporal dementia and parkinsonism linked to chromosome 17 (FTDP-17), and rare unclassifiable tauopathies.

#### Pick’s disease

Pick’s disease (PiD) is a rare 3R tauopathy that usually presents clinically with features of FTD, such as deterioration of language, personality, and memory. While most cases are sporadic, a few familial cases have been linked to missense mutations in *MAPT* [34, 35]. Circumscribed focal cortical atrophy (“lobar atrophy”) is a striking neuropathological feature, often macroscopically severe in anterior frontal and temporal lobes, as well as medial and inferior temporal cortices. This “knife-edge” atrophy is associated with severe neuronal loss and gliosis, with spongiosis and astrogliosis in the cortical ribbon and secondary axonal loss in the subjacent white matter. The striatum, subthalamic nucleus, and the substantia nigra are variably affected [36].

**Fig. 1:**
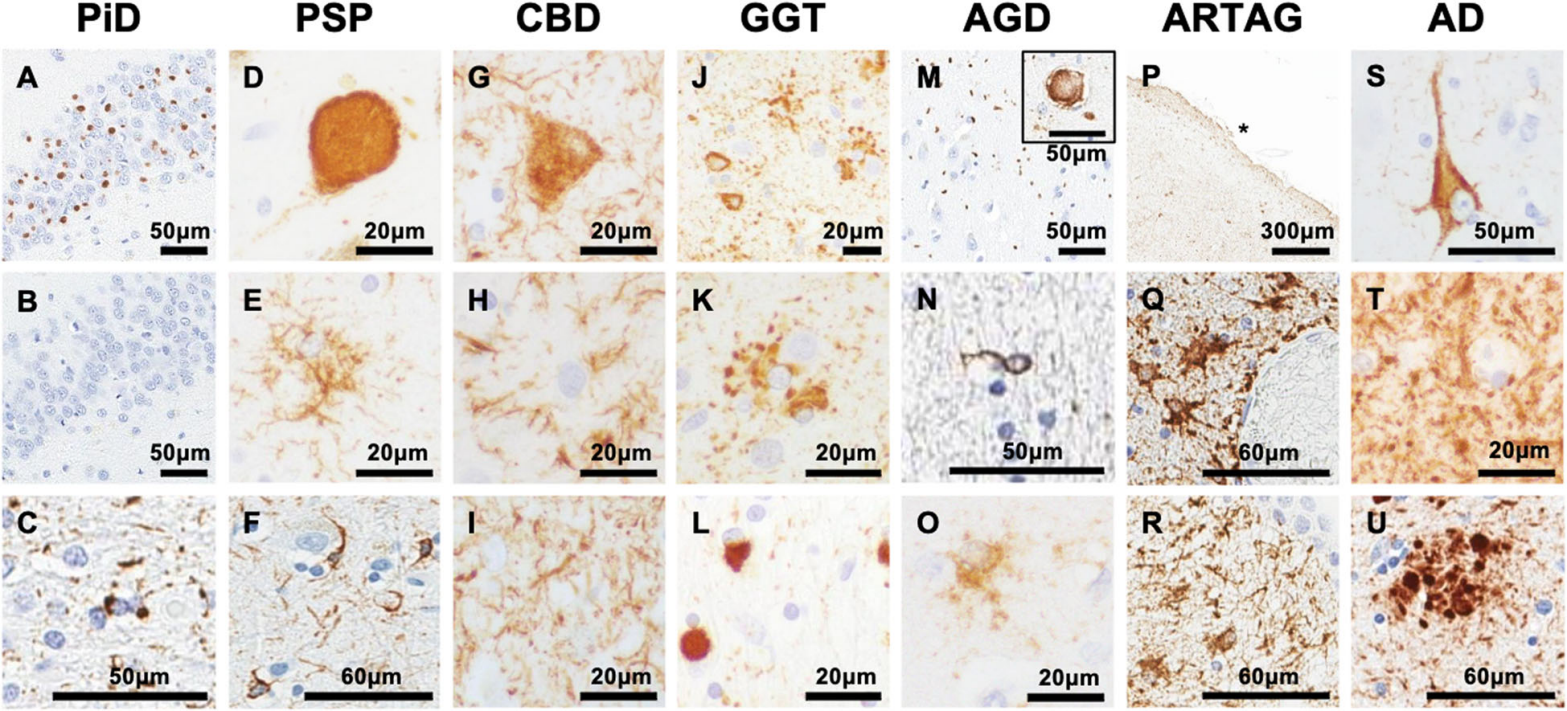
Various tau inclusions in primary tauopathies and AD. (**A-C**) PiD. Neuronal Pick bodies stain positive for 3R tau (**A**) while negative for 4R tau (**B**). White matter oligodendroglial inclusions (**C**). (**D-F**) PSP. Neuronal globose tangle (**D**). Tufted astrocyte (**E**). Oligodendroglial coiled bodies (**F**). (**G-I**) CBD. Ballooned neuron (**G**). Astrocytic plaque (**H**). Neuropil tau threads (**I**). (**J-L**) GGT. GAIs (*upper right*) and neuronal pretangles (*lower left*) (**J**). Higher magnification of GAIs (**K**). GOIs (**L**). (**M-O**) AGD. Argyrophilic grains (inset shows ballooned neuron) (**M**). White matter oligodendroglial coiled body (**N**). Bush-like astrocyte (**O**). (**P-R**) ARTAG. Thorn-shaped astrocytes in subpial (**P**), perivascular (**Q**), and subependymal regions (**R**). (**S-U**) AD. NFT (**S**). Neuropil tau threads (**T**). Dystrophic neurites in a neuritic plaque (**U**). Immunoreactivity to RD3 (3R tau) (**A**), RD4 (4R tau) (**B, M-O**), and CP13 (pS202 tau) (**C-L, P-U**). Asterisks denote the crest of the gyrus

PiD has prominent neuronal lesions in the form of ballooned neurons (or “Pick cells”) and Pick bodies. Ballooned neurons are not specific to PiD and can be seen in other tauopathies such as CBD and argyrophilic grain disease (AGD). The neuroanatomical distribution of Pick bodies parallels brain atrophy. They are well-circumscribed, round, neuronal cytoplasmic inclusions that are immunoreactive for p-tau and 3R tau (Fig. 1 A), but negative for 4R tau (Fig. 1 B). Pick bodies have variable (mostly negative) staining with the Gallyas silver impregnation method, and they are weakly positive or negative with thioflavin-S fluorescent microscopy [36, 37]. Of note, Pick body-like inclusions in hippocampal dentate gyrus neurons in AD [38, 39] differ from Pick bodies in histologic properties (argyrophilic and thioflavin-S-positive) and tau isoform composition (3R and 4R tau-positive).

Glial lesions are less frequent than neuronal lesions in PiD, but Pick-body-like inclusions can be detected in oligodendrocytes in affected white matter (Fig. 1 C) [36, 37, 40]. Biochemically, insoluble tau protein in PiD is enriched in 3R tau, but immunohistochemistry with antibodies specific to 4R tau often reveals sparse astrocytic lesions in most cases [37, 40]. As in all neurodegenerative disorders, neuronal loss and astrogliosis in PiD are accompanied by microgliosis. A common marker of activated microglia, HLA-DR, shows prominent microgliosis in the neocortex and white matter of PiD, including areas with sparse tau pathology [36]. Of note, the cortex and white matter contain only few lipid-laden amoeboid microglia, which may reflect a chronic disease process rather than distinct microglial activation as described in FTLD without tau pathology [41].

#### Progressive supranuclear palsy (PSP)

Progressive supranuclear palsy (PSP) is a sporadic 4R tauopathy with less than 5 % of patients having a family member with reported clinical history consistent with PSP [42]. Major clinical features of PSP include L-DOPA non-responsive parkinsonism, oculomotor dysfunction that significantly affects vertical eye movement, gait failure, and postural instability that is frequently accompanied by falls [43, 44]. The PSP syndrome has been reported in patients with damaging mutations in *MAPT* [45], but there are only a few autopsy-proven cases of familial PSP due to *MAPT* mutations, including two brothers with early-onset PSP and a *MAPT* p.S285R mutation [46]. The pathology of other *MAPT* mutations clustered in exon 10 and its splicing region (p.ΔN296, p.N296N, p.P301L, p.G303V, p.S305S, IVS10 + 3, and IVS10 + 6), which may alter the ratio of 3R and 4R tau, fits better with FTLD-tau than PSP [47, 48].

The tau gene has common variants that define two haplotypes, H1 and H2, associated with a large genomic inversion on chromosome 17 that includes the *MAPT* gene [49]. Most PSP patients carry the H1 haplotype, but several other non-tau genetic risk loci have been identified in a genome-wide association study (GWAS) of autopsy-confirmed PSP [50]. Intriguingly, the ε2 allele of apolipoprotein E (*APOE*) gene is potentially linked to increased risk of PSP independent of the H1 haplotype [51], although *APOE* was not identified as a risk allele in the PSP GWAS [50].

Histopathologically, PSP is characterized by neuronal and glial tau pathology, neuronal loss, and fibrillary astrogliosis, with the most severe neuronal loss found in the globus pallidus, subthalamic nucleus, and substantia nigra [52]. Globose NFT (Fig. 1D) or pretangles are frequent in these affected brain regions. The morphology of glial tau pathology (especially astrocytic) distinguishes PSP from other tauopathies [53]. The most characteristic glial lesion in PSP is the tufted astrocyte (Fig. 1E), but oligodendrocytes are also vulnerable (“coiled bodies”) (Fig. 1 F). In addition to vulnerable subcortical regions, the premotor and motor cortices, the ventral thalamus, corpus striatum, and the olivopontocerebellar output and input systems are also affected. Interestingly, unlike reactive astrocytes, tau-immunoreactive tufted astrocytes have minimal labeling with glial fibrillary acidic protein (GFAP) [54]. Moreover, the distribution of tufted astrocytes does not parallel the reactive astrogliosis in PSP, which is greatest in the aforementioned nuclei. Instead, tufted astrocytes are more frequent in the neocortex and striatum, where neuronal loss is less, suggesting that tufted astrocytes represent an independent degenerative disease process [54].

#### Corticobasal degeneration (CBD)

Corticobasal degeneration (CBD) is a sporadic 4R tauopathy with only a few *MAPT* mutations reported in rare familial cases (e.g., p.G389R and p.N410H) [55, 56]. Similar to PSP, CBD risk is strongly associated with the *MAPT* H1 haplotype [57]. An association with the *APOE* ε2 allele has been reported, but it did not reach a statistical significance [51]. CBD shows a wide range of clinical symptoms, including impaired execution of skilled movements (apraxia), parkinsonism, executive dysfunctions, language impairments, and even PSP-like features such as postural instability and gaze palsy [58]. At a neuroanatomic level, cerebral cortex and basal ganglia are preferentially affected in CBD as its name suggests, unlike in PSP in which the basal ganglia, subthalamic nucleus, and midbrain are disproportionately impacted [59].

A key histopathological feature of CBD is the ballooned neuron (Fig. 1G). Ballooned neurons are swollen neocortical neurons that contain phosphorylated neurofilament protein [60], but are also labeled with antibody to alpha-B crystallin [61]. Importantly, CBD shares similarities with PSP in its prominent accumulation of 4R tau in both neurons and glial cells. Astrocytic tau pathology in CBD is distinct from that in PSP in terms of morphology and anatomical distribution. Unlike tufted astrocytes in PSP, tau-positive astrocytes in CBD appear as annular clusters of astrocytic cell processes, which have been named “astrocytic plaques” [62] given their resemblance to neuritic plaques centered around Aβ in AD [59, 63]. While tufted astrocytes in PSP show tau immunoreactivity concentrated in cytoplasm and proximal astrocytic cell processes, astrocytic plaques in CBD show tau immunoreactivity concentrated in distal astrocytic cell processes (Fig. 1 H) [59, 63]. These morphological differences in astrocytic tau lesions in PSP and CBD represent distinct subcellular compartmentalization of pathological tau in astrocytes [64]. Oligodendroglial coiled bodies may also be found in CBD, but are less prominent than in PSP. Another prominent pathological feature of CBD is tau accumulation in neuronal processes as neuropil threads in affected gray and white matter of cortical and subcortical regions (Fig. 1I). Neuropil threads are also frequent in AD, where the term was originally used [65]; however, the neuroanatomical distribution of threads is different, with corticolimbic gray matter vulnerable in AD, while threads in CBD are in both gray and white matter of cortical and subcortical structures.

#### Globular glial tauopathy (GGT)

Globular glial tauopathy (GGT) is a rare non-familial 4R tauopathy; only a few individual cases have been linked to *MAPT* mutations [66–68]. A subset of GGT (Type 2) overlaps with an atypical form of PSP [69], while other types (Types 1 and 3) are distinctly different [70]. The major histopathological hallmarks of GGT are globular, tau-positive inclusions in astrocytes and oligodendrocytes that are enriched in 4R tau (Fig. 1 J). They are referred to as globular astrocytic inclusions (GAIs) and globular oligodendroglial inclusions (GOIs), respectively (Fig. 1 K-L). GOIs are argyrophilic on silver stains (e.g., Gallyas), while GAIs are negative or only weakly argyrophilic, which distinguish astrocytic lesions in GGT from those in PSP and CBD. The clinical spectrum of GGT includes features similar to those of FTD or motor neuron disease (MND) or both, leading to the classification of three different GGT subtypes [71]. In each GGT subtype, FTD-like symptoms reflect tau pathology affecting frontal and temporal cortices, while MND-like symptoms correspond to involvement of motor cortex, corticospinal tract degeneration [71]. While Type 2 GGT shows similarities with PSP, other subtypes (Types 1 and 3) are distinct frontotemporal tauopathies clinically and morphologically. In addition, recent biochemical data demonstrates striking differences in seeding potency of GGT brain lysates in cell-based reporter assays compared to brain lysates of other tauopathies such as PSP, CBD, and AD. This strongly supports the concept that GGT is a distinct tauopathy [29]. Recent biochemical characterization of tau in GGT shows distinct templating properties of 4R tau (with RT-QuIC assays [72]) in GGT compared to PSP and other 4R tauopathies. In addition, expression levels of aquaporin-4 and glial glutamate transporter were altered in GGT, suggesting potential unique pathologic mechanisms of oligodendroglial and astroglial dysfunction in GGT [73].

#### Argyrophilic grain disease (AGD)

The term “argyrophilic grains” were originally coined by Braak and Braak to describe numerous spindle-shaped profiles scattered in the neuropils of demented patients who lacked AD tau pathology [74]. These profiles appeared to be strongly positive on Gallyas silver stains, hence given the name “argyrophilic,” indicating their high affinity to silver. Argyrophilic grains were later found to be 4R tau-positive (Fig. 1 M), defining argyrophilic grain disease (AGD) as a 4R tauopathy [75]. Genetic studies have revealed that AGD patient have overrepresentation of the *MAPT* H1 haplotype like PSP and CBD [75], but no clear association with *APOE* [76].

Clinically, AGD can be associated with neuropsychiatric symptoms (e.g., personality changes and emotional instability) and memory impairment [75], but AGD is not always associated with cognitive impairment [77]. Cognitively unaffected individuals with AGD tend to show more restricted pathology in the ambient gyrus and the anterior portion of hippocampal CA1, while demented AGD patients have a more widespread pattern [77]. In addition to argyrophilic grains, ballooned neurons are frequent in the amygdala and limbic cortices (Fig. 1 M, inset). 4R tau-positive oligodendroglial coiled bodies, another feature of AGD (Fig. 1 N), are often limited to the white matter of the limbic lobe, while grains are mainly found in the neuropil of the entorhinal and perirhinal cortices, the hippocampus, and the amygdala. There is no specific astrocytic tau lesion assigned to AGD, yet tau-positive astrocytes often show a ramified to bushy appearance (Fig. 1O) [78].

#### Primary age-related tauopathy (PART)

Primary age-related tauopathy (PART) encompasses neuropathological changes that were previously regarded as a part of normal aging or neurofibrillary tangle predominant senile dementia [79]. PART is characterized by AD-like NFTs without amyloid co-pathology [80]. Specifically, “definite PART” is defined by the low Braak NFT stage (II-III), in which NFTs are frequent in the limbic system, including CA2 region of the hippocampus, amygdala and medial temporal lobe. The term “possible PART” is intended to indicate cases with similar NFT pathology, but also mild amyloid co-pathology. PART can overlap with some types of FTLD-tau. The most frequent *MAPT* mutations with limbic predominant NFT pathology are p.R406W and p.V337M [81]. Clinically, definite PART can be seen in those who were cognitively normal or those with cognitive impairment, especially amnestic syndromes. Cognitive impairment is more frequent at older ages (> 80 years) and in those with a positive family history of cognitive impairment [82]. In contrast to other primary tauopathies, most tau pathology in PART is neuronal [80].

Biochemical and ultrastructural studies have shown that NFTs in PART are positive for both 3R and 4R tau. Electron microscopy reveals a structural composition of PHFs, reminiscent of NFT pathology in AD. Despite these similarities, PART is not associated with *APOE4* [80]. That PART is an age-associated medial temporal tauopathy distinct from AD remains controversial[83].

#### Aging-related tau astrogliopathy (ARTAG)

While astrocytic tau lesions are characteristic of neurodegenerative tauopathies such as PSP, CBD, and GGT, they are also observed in brains of neurologically normal elderly individuals. Aging-related tau astrogliopathy (ARTAG) is defined by the presence of 4R tau-positive thorn-shaped astrocytes in subpial (Fig. 1P), perivascular (Fig. 1Q), and subependymal regions (Fig. 1R) [84]. Another pathologicalsubtype of ARTAG is tau-positive granular/fuzzy astrocytes in the gray matter, especially in the amygdala [84]. Thorn-shaped astrocytes in ARTAG can be distinguished from tufted astrocytes in PSP based on their co-immunoreactivity for GFAP [40, 84]. Astrocytic lesions in ARTAG share similarities with those in chronic traumatic encephalopathy (CTE). Yet, tau lesions in CTE have a predilection for the depths of the cerebral sulci in an unpredictable distribution [84], whereas they show a characteristic distribution and progression in ARTAG [85].

ARTAG is rarely observed in individuals younger than 60 years of age. It is rarely an isolated finding, but it is a common co-pathology. ARTAG is detected in more than 65 % of primary tauopathies [85]. While the etiology and clinical significance of ARTAG are poorly understood, studies have shown that ARTAG is associated with a significantly elevated level of another astrocytic protein aquaporin-4 [86], the major water channel in the brain. This might suggest that dysfunction of the blood-brain barrier (BBB) may at least partially contribute to the pathogenesis of ARTAG [85].

### Secondary tauopathies

#### Alzheimer’s disease (AD)

Alzheimer’s disease (AD), the most common form of dementia, is classified as a secondary tauopathy as its neuropathological diagnosis requires both amyloid deposition and tau aggregation [87]. Tau lesions in AD include NFTs (Fig. 1 S) and neuropil threads (Fig. 1T) composed of PHFs and straight filaments (SFs) that are immunoreactive for both 3R and 4R tau [87]. NFTs are the mature form of neuronal tau pathology, while pretangles represent an earlier stage of pathologic neuronal tau accumulation. In AD, NFTs have a stereotypic progressive pattern of distribution that allows for staging of AD-type tau pathology into six different stages that correlate with the progression of cognitive impairment [65, 88]. This staging scheme, which was proposed by Braak and Braak [65], suggests that the NFT pathology first develops in the transentorhinal cortex and subsequently appears in the limbic system, including the hippocampus and amygdala, ultimately affecting the isocortical regions, with primary cortices (e.g., primary visual cortex) involved in the most advanced disease stages. When neurons containing NFTs die, insoluble tau filaments remain in the extracellular space as “ghost tangles” [89]. Neuropil threads, composed of tau accumulation within dendrites and axons, also progress in association with the NFT distribution pattern. Moreover, a subgroup of neuritic plaques contains filamentous tau with dystrophic neurites surrounding amyloid deposits ("neuritic plaques") (Fig. 1U) [87]. Unlike diffuse Aβ plaques that lack neuritic elements, neuritic plaques are associated with cognitive deficits.

Tau-positive glial lesions are not a feature of AD unless there is a comorbidity with other tauopathies such as ARTAG or AGD. On the other hand, reactive astrocytes and activated microglia are frequently detected in affected brain regions. These aberrant glial responses are associated with neuritic plaques, indicating a significant contribution of neuroinflammation in AD.

### Tauopathies with environmental exposure

#### Chronic traumatic encephalopathy (CTE)

Chronic traumatic encephalopathy (CTE) is a sporadic tauopathy associated with repetitive traumatic brain injuries and related (sub)concussions. As such, contact sport players and military veterans are often reported as the most vulnerable populations [90, 91]. Recently, a retrospective study revealed that approximately 6 % of a population-based autopsy cohort (athletes and non-athletes combined) had pathology of CTE, and a history of playing contact sports; American football was notably associated with increased risk of pathology of CTE [92]. In a large referral autopsy series (Boston University Chronic Traumatic Encephalopathy Center), subjects with CTE pathology often had attention and memory deficits, as well as personality changes that were progressive [90]. A subset of CTE patients has also been reported to be associated with MND-like symptoms, showing muscle atrophy and speech impediment [93]. Genetic assessment has shown that there is no clear association between *APOE* genotype and the risk of developing CTE [94, 95].

**Fig. 2:**
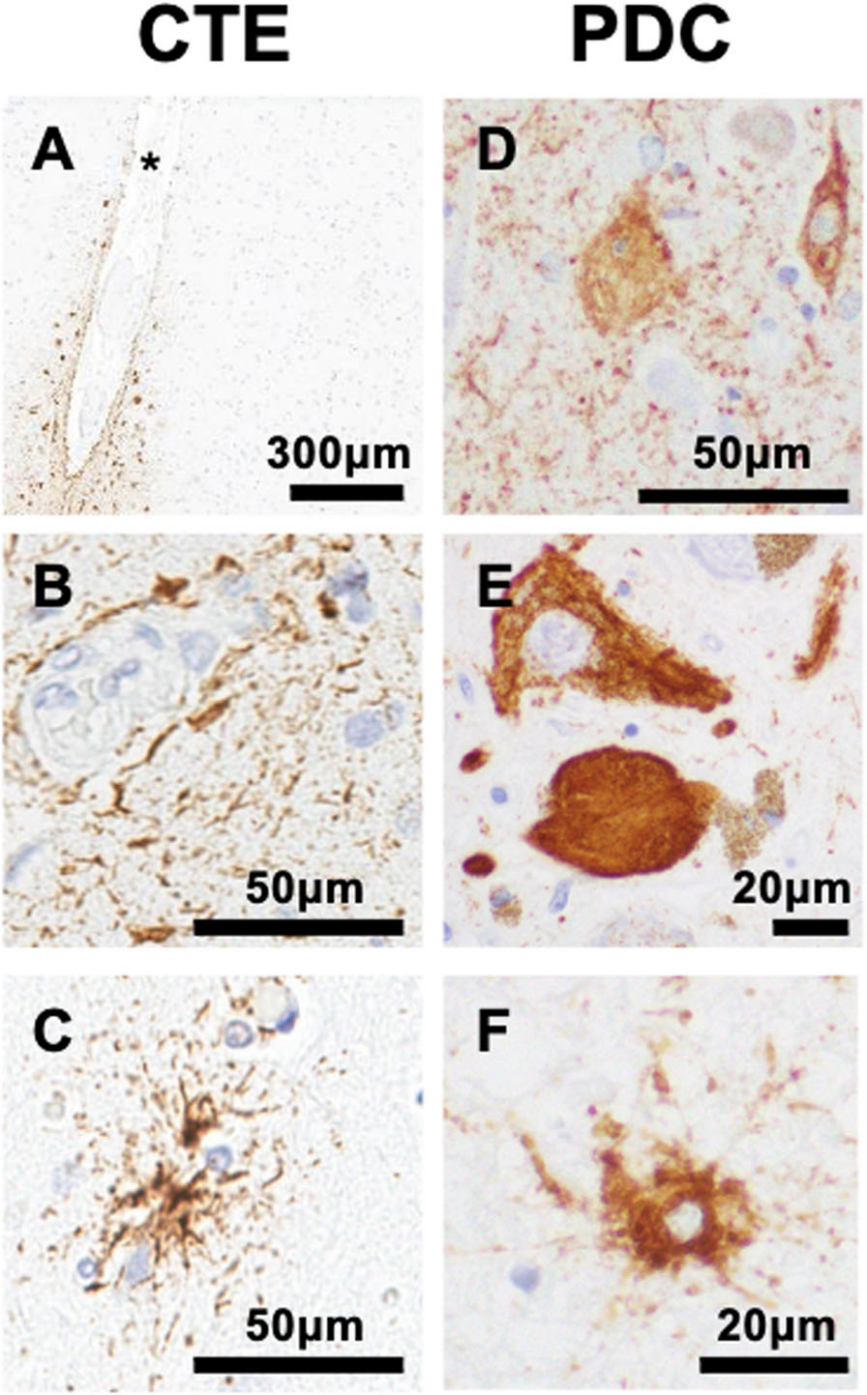
Various tau inclusions in multi-factorial tauopathies and AD. (**A-C**) CTE. Subpial tau at the depth of the cortical sulcus (**A**). Patchy perivascular tau (**B**). Thorn-shaped-like astrocytes (**C**). (**D-F**) Guam PDC. Tau threads, extracellular tangle, and NFT (**D**). Midbrain NFT and globose tangle (**E**). Ramified astrocytes in periaqueductal gray matter (**F**). Immunoreactivity to CP13 (pS202 tau). Asterisks denote the crest of the sulci.

Like AD, neuronal tau inclusions in CTE are positive for both 3R and 4R tau [96]. Unlike the predictable NFT distribution in AD, tau pathology in CTE is seemingly random, but thought to initiate at the depths of the sulci (Fig. 2 A), usually in the form of perivascular neuronal and glial tau lesions (Fig. 2B) [94]. The pathology of CTE needs is to be distinguished from ARTAG, which can also be detected in subpial spaces at the depths of cerebral sulci. Unlike ARTAG, CTE pathology is characterized by neuronal tau pathology at the depths of cortical sulci [97] (Fig. 2 C). At the advanced disease stage, tau pathology in CTE is found in most cortical regions, including medial temporal lobe. Progressive involvement of basal ganglia and brainstem is accompanied by pronounced brain atrophy [94]. Additionally, diffuse Aβ plaques can be detected in a subset of CTE, especially those individuals with more advanced age [94].

#### Geographically isolated PSP-like tauopathies

Guam Parkinsonism-dementia complex (PDC) is a geographically isolated tauopathy found in the Chamorro population of Guam in the Western Pacific, which was reported to be prevalent in the mid-1950’s (~ 1 in 400) [98]. While no *MAPT* mutations have been reported in Guam PDC, environmental factors such as toxins from cycad are debated to play a role in the pathogenesis, at least partially [99]. Guam PDC patients show several motor symptoms (rigidity, tremor, bradykinesia) as well as memory deficits and personality changes [98]. Neuropathological features of Guam PDC include cortical atrophy and depigmentation in the substantia nigra and locus ceruleus [98]. Similar to AD, Guam PDC exhibits 3R- and 4R-positive NFT pathology extensively distributed in the neocortex, hippocampus, and brainstem, but mostly in the absence of senile plaques (Fig. 2D, E) [98]. Neuropil threads are also observed (Fig. 2D) [98]. Both gray and white matter are affected by tau pathology, and unlike AD, glial tau inclusions are often detected in Guam PDC in astrocytes (Fig. 2 F) and oligodendrocytes [98].

Similar to Guam PDC, Guadeloupean parkinsonism represents a cluster of tauopathy that was prevalent among residents of the Guadeloupe islands in the French West Indies in the late 1990's [100, 101]. Clinically, these individuals displayed an atypical Parkinson syndrome with PSP-like features such as postural instability and vertical gaze impairment [100, 101]. Histopathological analysis of several cases revealed pre-tangles, NFTs, and threads composed of hyperphosphorylated tau in multiple brain regions including the midbrain, the striatum, and the cortex, while one case in particular showed occasional glial tau inclusions in the caudate and the putamen [101]. The etiology of Guadeloupean parkinsonism is speculated to be associated with the regional practice of consuming herbal tea and tropical fruits containing alkaloid toxins that are potent inhibitors of mitochondria [100, 101]. In addition to consumption of herbal toxins, exposure to industrial metals may contribute to the onset of tauopathies as reported in the small town in Northern France. Specifically, from 2007 to 2014, a spike of PSP-like tauopathy was observed (~ 12 times more than expected) in the Northern regions of France where soils are highly contaminated with arsenic and chromate from chemical plants [102]. Clinical and neuropathological presentations of this tauopathy were reminiscent of PSP; patients often showed gait and gaze abnormalities, and both NFT and tufted astrocytes were detected at autopsy [102].

## Dynamics between glial cells and tau pathophysiology

Functions of neuronal tau have been extensively investigated in various aspects of CNS physiology, including microtubule dynamics [103], axonal transport [104, 105], and neurite formation [106, 107]. Compared to neuronal tau, however, roles of glial tau remain to be further explored. The presence of robust glial tau inclusions in primary tauopathies highlights a crucial role of a primary degenerative process in astrocytes and oligodendrocytes that is likely amplified by a secondary glia-mediated neuroinflammation [108].

### Astrocytic functions and tau

Astrocytes, critical in supporting synaptic and neuronal metabolic functions, may play a dual role by either becoming a target of the degenerative process or contributing to the progression of neurodegeneration in their reactive state [109]. Tau astrogliopathy is a shared phenomenon across a number of primary tauopathies, yet its form and degree vary in a disease-specific manner. In addition to nonspecific reactive astrocytosis and dysregulation of astrocytic functions (e.g., glutamate transporter), astrocytes also accumulate disease-specific tau pathology [110]. Reactive astrocytes are well-documented in AD and other degenerative disorders [84, 111]. Reactive astrocytes, marked by immunoreactivity for astrocyte-specific intermediate filament, glial fibrillary acidic protein (GFAP), are associated with thorn-shaped astrocytes in ARTAG, but not with tufted astrocytes in PSP, suggesting that different astrocytic tau lesions occur through distinct mechanisms that may involve neuroinflammation [54, 84]. While still being actively investigated, several studies have demonstrated that reactive astrocytes can be triggered by inflammatory cytokines, complement component 1q (C1q), or mitochondria fragments secreted by microglia or astrocytes themselves [112, 113]. In addition to reactive astrocytosis, several key astrocytic proteins that are important in water and glutamate transport are altered in tauopathies with prominent astrocytic pathology, including GGT and CBD [114, 115]. It remains to be determined if aberrant changes in water homeostasis and glutamate transport contribute to accumulation of tau in astrocytes, or vice versa.

In addition to regulating the BBB, modulating inflammation, and aiding neuronal function, astrocytes can phagocytose synapses and other debris [116]. *In vitro* studies show astrocytes can mediate Aβ clearance by phagocytosis [117], and several studies have similarly demonstrated that astrocytes can also take up and degrade pathological tau species from the extracellular space [118, 119]. Interestingly, the transcription factor EB (TFEB), a master transcriptional regulator of lysosomal biogenesis, was found to enhance the efficiency of astrocytic uptake and clearance of tau in both the primary cultures and mouse models [118]. Another study suggested that astrocytes can internalize tau monomers via pathways mediated by heparan sulfate proteoglycans (HSPGs), a transcellular tau propagation mechanism initially described for neurons [119]. While further studies are required to determine if other mechanisms can also mediate astrocytic uptake of tau, it has been speculated that astrocytic tau inclusions are the result of tau seeds that were internalized by astrocytes, but failed to be degraded. A similar possibility has been tested for Aβ in a study that showed Aβ, engulfed by astrocytes, can accumulate to form inclusions resistant to intracellular degradation [120]. Although it remains to be determined if external sources account for tau in astrocytic lesions, it is also possible that tau in astrocytic lesions is intrinsic to astrocytes, reflecting astrocytic dysfunction and degeneration. Importantly, in addition to displaying tau inclusions, astrocytes have been reported to facilitate Aβ-induced tau phosphorylation in neurons [121], indicating that their role in modulating tau pathology can be multifaceted.

Additional studies are warranted to elucidate innate or acquired aberrant functions of astrocytes that may potentially promote neurodegeneration. For instance, a recent genome wide association study in FTLD-tau revealed that the *TBKBP1* gene (TANK-binding kinase 1-binding protein) is important in innate immune response. This gene is enriched in fetal astrocytes [122], which suggests that it might have relevance to astroglial tauopathy. Additional studies are needed to explore the link between an increased risk of primary tauopathies and disrupted astrocytic immune responses.

### Oligodendrocytic functions and tau

Oligodendrocytes are the key source for myelin that form sheaths around axons in the CNS, and they also express tau [6]. Several studies using mouse models have shown that oligodendroglial tau can facilitate myelination by inducing myelin basic protein and promoting outgrowth of oligodendrocyte processes that wrap around axons [123–125]. Hence, correct sorting of tau into oligodendroglial processes is believed to be critical for tau-mediated myelination [126]. Oligodendrocyte dysfunction can lead to dysmyelination, causing subsequent degeneration of white matter [127]. In fact, structural abnormalities of white matter in AD have been reported using antemortem neuroimaging [127, 128]. Significant degeneration of white matter has also been noted in primary tauopathies such as PSP [129], CBD [130], and GGT [71].

Tau inclusions in oligodendrocytes can appear as coiled bodies in several tauopathies including PSP, CBD, and AGD, as well as globular Pick body-like inclusions in PiD and GOIs in GGT [131]. On the other hand, oligodendroglial tau lesions are not prominent in AD, PART, ARTAG, or CTE [131]. Given that loss-of-function of oligodendroglial tau can compromise myelination [125], a crucial link may exist between pathological tau aggregates in oligodendrocytes and white matter degeneration in tauopathies. Interestingly, a recent study has shown that injection of tau seeds directly into the lateral corpus callosum of WT mice can lead to aggregation of mouse endogenous tau in both ipsilateral and contralateral corpus callosum, frequently associated with myelin disruption [132]. In this study, oligodendroglial tau lesions induced by tau seeds from various tauopathy brain samples were reminiscent of coiled bodies of PSP, AGD and CBD [132]. As such, oligodendrocytes and white matter may be involved in tau propagation in tauopathies.

### Microglial functions and tau

Microglia, the brain-resident macrophages involved in neurodevelopment, regeneration, and neuroinflammation [133], maintain surveillance during homeostasis [134]. In neurodegenerative conditions, microglia react by mediating clearance of pathological protein aggregates as demonstrated by *in vitro* studies [133]. Importantly, neurodegenerative processes can be promoted by microglial dysfunction, which leads to impaired damage repair and aberrant neuroinflammatory responses [133]. Microglial activation is a notable feature in all tauopathies, but the initial trigger and efficiency of clearing pathologic tau aggregates likely vary. In AD, multiple studies have demonstrated that activated microglia are closely associated with neuritic plaques and ghost tangles of insoluble tau filaments [87]. Genetic evidence from a series of AD GWAS [135–141], as well as the more recent GWAS [142, 143], further suggests a strong role for microglia in pathogenesis of AD. Similar genetic evidence is lacking in primary tauopathies such as PSP and CBD, even though enhanced microglial activation has been shown to correlate with severity and distribution of tau pathology [144].

Unlike oligodendrocytes and astrocytes, microglia do not express tau, although a few reports have shown immunohistologic evidence of co-localization of tau and microglia [145–147]. It remains uncertain if these are microglial tau inclusions. Internalization of tau by microglia has been supported by several studies that have highlighted potential involvement of microglia in exosome-dependent tau propagation [148, 149] or an asparagine endopeptidase-mediated tau cleavage [150]. Moreover, multiple studies have demonstrated the importance of microglia-mediated neuroinflammation and phagocytosis in modulating tau pathology by manipulating microglia or their receptors in cell culture and animals (extensively covered by Leyns and Holtzman [108]).

## Lessons from transcriptomics

In addition to animal studies that extend our understanding of mechanisms underlying tau pathology in different cell types, transcriptomic approaches have been highly valuable, especially when assessing data directly obtained from human brain tissues. A recent transcriptome-wide association analysis of gene expression levels in PSP brain tissues determined whether cell type-specific tau pathology in PSP can be derived from transcriptional changes in the brain [151]. This analysis revealed a highly unique pattern of transcripts and expression networks that were associated with each cell type-specific tau lesion. In particular, NFT pathology was positively associated with a brain co-expression network enriched for synaptic and PSP candidate risk genes, while negatively associated with immune system transcripts. Conversely, tufted astrocytes were negatively associated with synaptic genes, but positively associated with the immune system and enriched for microglial genes. These findings suggest that aberrant immune transcript expression may specifically underlie the astrocytic tau pathology in PSP, while they are not clearly associated with NFT. Intriguingly, these data are consistent with microglial activation in systems severely impacted in PSP such as pyramidal, extrapyramidal, and cerebellar output systems [54]. The lack of evidence that tufted astrocytes are associated with astrocytic genes also corresponds to how tufted astrocytes are distinct from reactive astrocytes [54]. Taken together, contrasting patterns of transcriptional association between NFT and tufted astrocytes strongly suggest that distinct pathomechanisms drive cell type-specific tau pathology in PSP. It remains to be determined whether microglial activation in PSP is merely a reactive phenomenon or if it might also promote formation of glial tau pathology.

Recently, single-cell (scRNA-Seq) and single-nucleus RNA sequencing (snRNA-Seq) techniques have greatly increased the resolution of transcriptomic analysis compared to conventional bulk RNA-Seq. This method extends the ability to identify distinct cell populations with unique transcriptomic signatures [152–155], and it has led to increased interest in determining cell type-specific response to pathological insults, including those in neurodegenerative diseases. For instance, one study interrogated two independent sets of snRNA-Seq data obtained from healthy human brain tissues and found that excitatory neurons have significantly elevated levels of aggregation-prone and “metastable” proteins, as well as genes that can enhance tau aggregation (“tau aggregation promoters”) [156]. These neurons had simultaneously decreased levels of genes that prevent tau aggregation (“tau aggregation protectors”), suggesting vulnerability to tau pathology. Conversely, the level of tau aggregation protectors was elevated in astrocytes, oligodendrocytes, and microglia. Combining this finding with the lower level of endogenous tau in glia, the study suggested that these factors can collectively contribute to the lack of glial tau pathology in AD. From these analyses, however, it can also be speculated that primary tauopathies with a more pronounced glial tau pathology are driven by unique pathomechanisms completely distinct from those of AD, which will be interesting to further investigate.

In addition to studies of healthy controls, several snRNA-Seq studies have analyzed AD brain tissues to reveal AD-associated transcriptional changes at a single-nucleus level [157–159]. Importantly, some of their findings suggested that differentially expressed genes (DEG) earlier in the AD pathogenesis were altered in a highly cell type-specific manner, highlighting the importance of evaluating different regulatory responses of each cell type to tau pathology. So far, no study has used snRNA-Seq to examine transcriptional landscape in primary tauopathies. Given that primary tauopathies have more robust glial tau pathology, it will be important to investigate cell type-specific transcriptional changes in tauopathies compared to AD. Understanding this aspect of tau-related pathomechanisms will greatly facilitate efforts to increase the efficacy of tau-targeted therapeutic strategies.

## Lessons from cryo-EM studies

The patient-based structural biology of tauopathies has recently witnessed one of the most exciting advancements in the field, powered by cryogenic electron microscopy (cryo-EM) [160] that uncovered the high-resolution atomic structure of insoluble tau filaments directly extracted from various tauopathies. In 2017, a seminal paper revealed that the insoluble core of tau fibrils from sporadic AD includes the last two repeats in the repeat domain (R3, R4) and a few residues beyond them (amino acid residues 306 to 378 of the longest tau isoform) [161]. A subsequent cryo-EM study also found the nearly identical tau core structure in both sporadic and familial AD cases [162], proposing a common tau fold for AD regardless of its genetic or environmental underpinnings. This core structure is unique to AD tau filaments as a novel fold of pathological tau filaments was reported for PiD (amino acid residues 254 to 378 of the 3R tau) [163], potentially due to different tau isoforms implicated in the lesion: 3R tau in PiD versus 3R and 4R tau in AD. Yet, the conformation of tau filaments in CTE was found to be distinct from that of AD, despite 3R and 4R tau pathology shared between two diseases [164]. A unique hydrophobic cavity in the CTE tau core further indicates potential incorporation of unidentified factors that may contribute to the CTE-specific tau aggregation [164].

Two recent cryo-EM studies have additionally discovered the unique conformation of CBD tau filament core that, like CTE, includes a hydrophilic cavity [165, 166]. Similar to CTE, an unknown cofactor was associated with the CBD tau core [165, 166], while its exact role in promoting CBD-specific tau aggregation is unknown. Both studies have consistently shown two types of CBD tau filaments made of an identical protofibril – one type with a single protofibril (“type I” [165] or “singlet” [166]) and the other including two protofibrils (“type II” [165] or “doublet” [166]). A combinatorial mass spectrometry-based proteomic analysis additionally found distinct ubiquitination and acetylation patterns in tau filaments core region between CBD and AD [166]. Interestingly, two types of CBD tau filaments also exhibited different ubiquitination and acetylation patterns [166], suggesting that specific PTM events occur not only between tauopathies, but also within the same tauopathy. A more recent study has expanded the investigation to identify structures of tau filaments in other 4R tauopathies: PSP, GGT, AGD, and ARTAG [167]. Similar to CBD-tau, this study also found multiple types of structurally different tau filaments in each tauopathy, indicating that pathological tau can possess distinct folds in the same disease [167]. Furthermore, this study revealed that tau filaments in an atypical PSP case with limbic globular 4R-positive neuronal inclusions had a unique structure intermediate between GGT and PSP tau filaments, providing molecular evidence to define this case as a novel class of tauopathy, which they termed as Limbic-predominant Neuronal inclusion body 4R Tauopathy (LNT) [167].

While cryo-EM studies have rapidly advanced our understanding of structural details of pathological tau filaments in various tauopathies, tau filament extraction methods used in these studies do not distinguish cell types from which insoluble tau is derived. For instance, multiple different types of tau protofibrils were observed in CBD, PSP, GGT, AGD, and ARTAG, but their cellular origin is mainly unknown as the bulk tissue has been used to extract tau fibrils [165–167]. Since both neuronal and glial tau lesions are prominent in these 4R tauopathies, whether each type of tau filaments is specific to a certain cell type remains to be determined. Potentially, techniques such as laser-capture microdissection can be applied to extract tau filaments in a cell type-specific manner, as this method allows isolation of specific types of cells from the frozen brain tissues [168]. Yet, much optimization will be needed to utilize such techniques for cryo-EM studies, as the analysis of the atomic structure requires a substantial amount of tau fibrils. While considering this option, examination of insoluble tau in other tauopathies, such as PSP and GGT, will further elucidate the link between certain structural properties of tau and disease-specific and/or cell type-specific tau aggregation. Ultimately, these findings will greatly aid the development of structure-based, disease-specific drugs that can efficiently target pathological tau species in each disease.

## Transgenic mouse models that reflect cell-type specific features of tau lesions

Since the identification of *MAPT* mutations in FTD [169–171], a myriad of transgenic mouse models has been developed to recapitulate tau aggregates and associated pathological changes in tauopathies (reviewed by Dujardin et al. [172]). Many of these mouse models show tau aggregates specifically in neurons, largely due to the use of neuron-specific promoters that drive the neuronal expression of transgene. Yet, glial tau pathology was reported in some of the earlier mouse models, such as TG23 mice (p-tau in astrocytes [173]) and G272V transgenic mice (oligodendroglial tau inclusions [174]) (Table 2). Moreover, models that have been previously characterized for their neuronal tau pathology were later shown to exhibit glial tau lesions. For instance, JNPL3 transgenic mice [175], overexpressing P301L human mutant tau, have tau inclusions in oligodendrocytes and astrocytes in addition to neuronal tau pathology [176]. These oligodendroglial tau inclusions, abundant in the white matter of the spinal cord and brainstem, resemble coiled bodies in human tauopathies. Similarly, another well-established tau transgenic mouse model, rTg4510 [177], was found to have oligodendroglial tau inclusions [178]. Interestingly, these inclusions were composed of endogenous mouse tau, suggesting that mouse tau can also form aggregates in glial cells. It is possible that glial tau pathology was not initially reported due to its lower abundance compared to neuronal pathology in these mouse models.

**Table 2:** A list of transgenic mouse models showing tau lesions in non-neuronal cell types

Name (if applicable)	Human tau isoform	Mutation (if applicable)	Promoter	Type of p-tau + cells	Ref.
**TG23**	0N3R	WT	HMG-CR	Neuron, astro	[173]
**-**	2N4R	p.G272V	mPrP-tTA	Neuron, olig	[174]
**JNPL3**	0N4R	p.P301L	mPrP	Neuron, astro, olig	[175, 176]
**rTg4510**	0N4R	p.P301L	CaMKII-tTA	Neuron, olig	[177, 178]
**Tα1-3RT tau Tg**	0N3R,1N3R, 2N3R	WT	mTα1α-tubulin	Astro, olig	[179]
**T-279**	2N4R	p.N279K	hTau	Neuron, astro	[180]
**GFAP/tauWT**	1N4R	WT	GFAP	Astro	[115, 181]
**GFAP/tauP301L**	1N4R	p.P301L	GFAP	Astro	[115]
**-**	1N4R	WT or p.P301L	mCNP	Olig	[182]

Abbreviations: *astro* astrocyte; *CaMKII* calcium/calmodulin kinase IIα promoter; *GFAP* Glial fibrillary acidic protein; *HMG-CR* HMG-CoA reductase; *hTau* human tau; *mCNP* mouse 2', 3'-cyclic nucleotide 3'-phosphodiesterase; *mPrP* mouse prion; *olig* oligodendrocyte; *tTA* tetracycline-responsive transactivator; *WT* wild-type

In 2002, the Tα1-3RT tau transgenic mouse model was developed to overexpress all three 3R tau isoforms in both neurons and glia, under the mouse Tα1 α-tubulin promoter [179]. Intriguingly, while lacking neuronal tau pathology, these mice developed glial tau inclusions similar to astrocytic plaques and oligodendroglial coiled bodies. In 2007, the T-279 mouse model expressing N279K mutant human tau recapitulated both neuronal tau inclusions and astrocytic tau inclusions, the latter reminiscent of tufted astrocytes in PSP [180]. Unlike many of tau transgenic mouse models that robustly overexpress the tau transgene, this model expresses human tau transgene at a very low level under the human tau promoter that regulates the transgene expression similar to the endogenous pattern. Accompanied by behavioral dysfunctions, such as impaired motor functions and spatial memory deficits, T-279 mice exhibited caspase-3 activation in neurons and astrocytes that are indicative of apoptotic cell death, shedding light on the potential molecular mechanisms underlying age-dependent neurodegeneration in these mice.

Efforts to recapitulate glial tau inclusions in a more controlled manner led to the development of mouse models that express human tau transgene under the astrocyte- or oligodendrocyte-specific promoter. For example, the GFAP/tauWT and GFAP/tauP301L mouse models were generated to express WT or P301L human mutant tau under the astrocytic GFAP promoter [115, 181]. Both mouse models progressively formed astrocytic tau lesions reminiscent of tufted astrocytes and astrocytic plaques, as well as neuropil thread pathology. These mice also showed impaired motor strength, which correlated with reduced expression of the glutamate transporter-1 (GLT-1), the mouse analogue of the human excitatory amino acid transporter 2 (EAAT2). Interestingly, the level of glial glutamate transporters has been shown to be substantially decreased in CBD, but not in AD, which lacks astrocytic tau pathology [115]. These mouse models, along with subsequent validation using post-mortem human brain tissues, suggested that disrupted glutamate homeostasis is potentially linked to astrocyte dysfunction in tauopathies that have robust glial tau pathology.

For oligodendrocyte-specific tau expression, one study developed mouse models expressing either WT or P301L mutant human tau under the CNP (2’,3’-cyclic nucleotide 3’-phosphodiesterase) promoter [182]. Among them, the PL transgenic line, with a high expression level of P301L tau, formed fibrillar tau inclusions in oligodendrocytes, preceded by impaired axonal transport and structural disruption of myelin and axon. Importantly, similar myelin abnormalities are also present in PSP and CBD. Moreover, both PSP and CBD share genetic risk factor in the *MOBP* gene (myelin associated basic protein) [183]. Along with the age-dependent loss of motor function, PL transgenic mice exhibited progressive degeneration of both oligodendrocytes and neurons, suggesting that oligodendroglial tau inclusions and degeneration may contribute to neurodegeneration.

Transgenic mouse models exhibiting astrocytic or oligodendroglial tau inclusions have facilitated our understanding of potential pathogenic factors involved in glial tau pathology in primary tauopathies. In particular, studies using mouse models in which tau transgene expression is confined to astrocytes or oligodendrocytes have served as valuable tools to elucidate previously unknown molecular links between glial tau lesions and various pathological features in primary tauopathies, as subsequently confirmed in human samples [115, 181, 182]. Characterization of mouse models, combined with biochemical and immunohistologic validation from post-mortem tauopathy brain tissues, as well as genetic evidence from human transcriptomic studies, may continue to help us investigate the pathogenesis of glial tau pathology and its impact on the pathology of primary tauopathies.

## Injection mouse models that recapitulate cell-type specific features of tau inclusions

In addition to conventional transgenic mouse models, sporadic mouse models have been recently developed to reflect pathological heterogeneity of tau inclusions (Table 3). As mentioned earlier, injection of seeding materials into the brains of WT or tau transgenic mice has been a popular approach to investigate *in vivo* tau seeding and propagation [19–28]. In particular, one study demonstrated that intracerebral injection of different tauopathy brain homogenates into transgenic mice expressing WT human tau can induce various types of neuronal and glial tau inclusions that closely resembled tau lesions characteristic to each tauopathy used in the injection materials [26]. Specifically, astrocytic lesions induced by PSP and CBD brain homogenates appeared morphologically similar to tufted astrocytes and astrocytic plaques, respectively. This intriguing finding suggested that tau species in primary tauopathies characterized by distinct glial inclusions have capacity to maintain cell type-specific propagation preference, providing another piece of evidence for the tau conformer hypothesis.

**Table 3:** A list of sporadic mouse models developed by injection of tau seeding materials

Genetic background	Injection sites & length	Injection materials	Type ofp-tau + cells	Type of tau lesions	Brain areas with lesions	Ref.
**ALZ17 (tg mice expressing WT human tau) (3-mo)**	HPC, CTX (for 6–15 months)	AD brain homogenates	Neuron, olig	NFTs, NTs, dystrophic neurites, coiled bodies	HPC and several connected areas (e.g., fim, amyg, thalamus, etc.); PiD-induced lesions were limited to injection sites	[26]
		TD brain homogenates	Neuron, olig	NFTs, NTs, coiled bodies		
		PiD brain homogenates	Neuron, olig	Short and thick NTs, coiled bodies		
		PSP brain homogenates	Neuron, olig, astro	Nerve cell body inclusions, NTs, coiled bodies, tufted astrocytes		
		CBD brain homogenates	Neuron, olig, astro	Nerve cell body inclusions, NTs, coiled bodies, astrocytic plaques		
		AGD brain homogenates	Neuron, olig, astro	Argyrophilic grains, NFTs, coiled bodies, non-fibrillar astrocytic pathology		
**Non-tg mice (C57BL/6; 2-3-mo)**	HPC, CTX (for 1–9 months)	Sark-insoluble AD-tau	Neuron	Pretangles	HPC, CTX	[28]
		Sark-insoluble PSP-tau	Neuron, olig, astro	Mature tangles, coiled bodies, tufted astrocytes, astrocytic plaques	HPC, CTX, fim, CC	
		Sark-insoluble CBD-tau	Neuron, olig, astro	Mature tangles, coiled bodies, astrocytic plaques	HPC, CTX, fim, CC	
**Non-tg mice (C57BL/6; 3–7 mo)**	Ventricles, HPC (for 3–7 months)	Sark-insoluble ARTAG-tau	Neuron, olig, astro	p-tau + neurons/oligo/astro	HPC, fim, CC, fornix	[184]
**Non-tg mice (C57BL/6; 3- 12-mo)**	HPC, CC, and CPu (for 4–7 months)	Sark-insoluble GGT-tau	Neuron, olig	Pretangles, NTs, coiled bodies	HPC, fim, CC, CPu	[114]
**TauKDn**^**cre;fl/fl**^**(specific knockdown of neuronal mouse tau; age n/d)**	HPC, CTX (for 3–6 months)	Sark-insoluble AD-tau	Little to no p-tau + cells	No tau lesions	No tau lesions	[185]
		Sark-insoluble PSP-tau	Neuron, olig, astro	NTs, coiled bodies, tufted astrocytes	HPC, fim, CC	
		Sark-insoluble CBD-tau	Neuron, olig, astro	NTs, coiled bodies, astrocytic plaques,	HPC, fim, CC	
**6hTau (expressing all six human tau isoforms; no endogenous mouse tau; 3-5-mo)**	HPC, CTX (for 1–9 months)	Sark-insoluble AD-tau	Neuron	NFTs, NTs	HPC, CTX	[186]
		Sark-insoluble PSP-tau	Neuron, olig, astro	NFTs, NTs, p-tau + oligodendrocytes, tufted astrocytes	HPC, CTX, fim	
		Sark-insoluble CBD-tau	Neuron, olig, astro	NFTs, NTs, p-tau + oligodendrocytes, astrocytic plaques	HPC, CTX, fim, CC	
		Sark-insoluble PiD-tau	Neuron, olig	NFTs, NTs, p-tau + oligodendrocytes	HPC, CTX, fim	

Abbreviation: *AD* Alzheimer’s disease; *AGD* argyrophilic grain disease; *amyg* amygdala; *ARTAG* aging-related tau astrogliopathy; *astro* astrocyte; *CBD* corticobasal degeneration; *CC* corpus callosum; *CPu* caudate/putamen; *CTX* cortex; *DG* dentate gyrus; *fim* fimbria; *GGT* globular glial tauopathy; *HPC* hippocampus; *mo* month-old; *n/d* not described; *NFT* neurofibrillary tangles; *NT* neuropil thread; *olig* oligodendrocyte; *PiD* Pick’s disease; *PSP* progressive supranuclear palsy; *sark* sarkosyl; *TD* tangle-only dementia; *tg* transgenic

This notion has been supported by a more recent study that used a similar approach by intracerebrally injecting pathological tau filaments isolated from human tauopathy brains (AD, PSP, CBD) into non-transgenic mice [28]. These “sporadic” tau mouse models displayed aggregation of endogenous mouse tau in the brain upon the injection of different tau filaments. Similar to previous reports, only PSP-tau and CBD-tau, but not AD-tau, induced robust and distinct astrocytic and oligodendroglial tau lesions. These mice also demonstrated time- and region-dependent transmission of glial tau pathology. The glial tau lesions continued to spread to specific parts of the brain over time. Importantly, PSP-tau induced more oligodendroglial tau inclusions, while CBD-tau induced more astrocytic tau inclusions. In the PSP-tau-injected mouse brains, oligodendroglial tau inclusions were observed to spread from the ipsilateral to contralateral white matter tracts, suggesting the potential contribution of either oligodendrocytes or myelinated axons (or both) in transmission of glial tau pathology. In the CBD-tau-injected mouse brains, the regions with a temporal increase in astrocytic tau lesions exhibited concomitantly decreased neuronal tau lesions, suggesting that glial tau lesions developed either more slowly compared to neuronal tau lesions, or upon receiving tau seeds released from neurons. Of note, several other studies have also demonstrated induction of various tau pathologies in non-transgenic mouse brains by injecting tau seeding materials prepared from various tauopathies, such as ARTAG and GGT [114, 184], further elaborating on how tau pathology can continue to propagate upon inoculation while maintaining cell-type specificity.

Several questions remain on the mechanisms underlying glial tau pathologic transmission, including whether the presence of neuronal tau is required to form glial tau aggregates. Recently, a mouse model termed TauKDn ^cre;fl/fl^ was developed to specifically knock down neuronal tau while preserving glial tau expression [185]. Upon intracerebral injection of CBD- or PSP-tau, these mice developed oligodendroglial tau pathology similar to the WT mice, indicating that oligodendroglial tau lesions do not require the presence of neuronal tau. Resulting tau lesions were mostly localized to the processes of oligodendrocytes, further supporting a potential oligodendrocyte-to-oligodendrocyte transmission mechanism. Unlike oligodendroglial tau lesions, astrocytic tau lesions failed to propagate in the absence of neuronal tau, suggesting the existence of distinct transmission mechanisms for oligodendroglial and astrocytic tau pathology.

Is the formation of cell type-specific tau pathology dependent on different tau isoforms? To explore this possibility, another recent study generated the 6hTau mouse model in which six WT human tau isoforms replaced mouse endogenous tau (predominantly 4R in adult brains) [186]. This model, expressing an equimolar ratio of 3R and 4R tau, was similarly injected with pathological tau fibrils isolated from AD, PSP, CBD, and PiD, which led to cell type-specific transmission of tau strains similar to previous studies. Specifically, AD-tau exclusively induced neuronal tau pathology, whereas PSP- and CBD-tau induced tau pathology in astrocytes and oligodendrocytes in addition to neurons. Of note, PiD-tau induced tau pathology mostly in neurons, but also in a subset of oligodendrocytes. This study suggests that tauopathy-specific tau strains can maintain their cell-type specificity during pathological transmission, independent of tau isoforms that respond to seeding materials.

Despite their more recent development, sporadic tau mouse models based on injection of seeding materials have been useful in investigating mechanisms and pathways implicated in cell type-specific tau lesions. While tau lesions in many of these mouse models recapitulated morphological similarities and anatomical distribution of aberrant tau pathology found in human tauopathies, not all pathological features were reflected in these models. For example, injection of tau fibrils from GGT brain tissues only resulted in tau lesions in neurons and oligodendrocytes, but not in astrocytes [114]. This discrepancy could be potentially due to differences in structures and isoform ratio between human and murine tau [114]. This demonstrates persistent challenges in developing mouse models that faithfully recapitulate pathological hallmark of human tauopathies. Still, efforts to create novel mouse models that more closely demonstrate abnormal tau lesions and associated pathology will continue to facilitate our understanding of pathomechanisms underlying tauopathies.

## Remaining questions for selective vulnerability in tauopathies

In this review, we summarized neuropathologic features and experimental findings that may help explain the selective vulnerability of the primary tauopathies, with a focus on glia. Yet, much remains to be understood with respect to what events can initially predispose, trigger, and propagate tau pathology in such a diverse manner. In particular, reports of tauopathies such as CTE and Guam PDC, which are possibly driven by specific environmental exposures further highlight the gap in our understanding of clinical, pathological, biochemical and cell biological heterogeneity of primary tauopathies.

While many of the neuronal lesions in different tauopathies share morphologic features in primary and secondary tauopathies, the regional vulnerability is different. Modeling anatomical vulnerably and disease progression in AD has been possible by assembling data from large autopsy series [65]. This is more challenging for the primary tauopathies, which are much less common, and possibly inherently more variable [43, 187]. A recent study suggested a staging scheme for PSP and concluded that, like AD, late-stage PSP involved the occipital cortices [122]. This remains to be confirmed, but more importantly, the scheme does not provide a clear anatomical framework linking early and late stages of PSP in terms of neuroanatomical connectivity. Except for CTE [94], there is a dearth of information on staging of primary tauopathies, and the sequential spreading of pathological tau still cannot explain the observed regional vulnerability in CTE, which exhibits strikingly uneven distribution of neuronal and glial pathology in cortical and subcortical regions. In AD, primary cortices such as the motor cortex, show relative resistance to tau pathology, mostly involved in the final disease stages. In contrast, the motor cortex is affected early in the disease course of PSP. Factors other than neural connectivity and tau spreading may contribute to selective vulnerability in tauopathies, but these factors currently remain unknown.

Regional differences in neuronal subtypes and networks are often used to explain selective vulnerability [188, 189], yet this phenomenon is largely understudied in neurodegenerative tauopathies. Most studies have examined glia as a uniform category and do not distinguish different subpopulations or their potentially diverse functionality. For example, oligodendroglia in cortical gray matter likely differ from those in white matter in terms of selective vulnerability given considerable differences in the microenvironment of cortical gray matter and myelinated white matter. To better understand cellular and pathological heterogeneity of tau lesions, it will be critical for future studies to address the potential role of glial diversity in pathogenesis of tauopathies.

## Concluding remarks

**Fig. 3:**
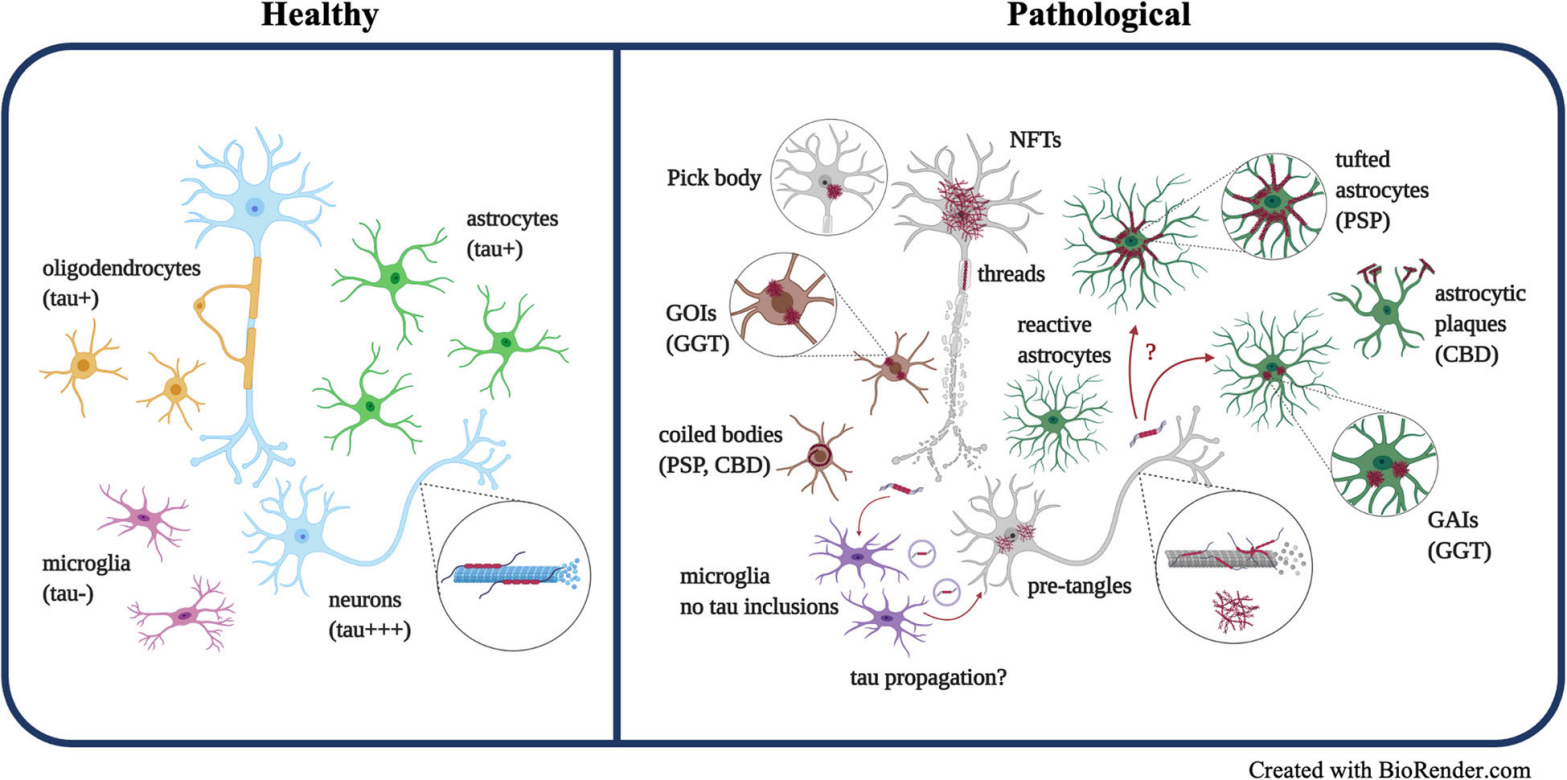
An illustration depicting a range of pathological tau lesions in different cell types in tauopathies. In the healthy brain (*left*), microtubule-binding protein tau interacts with neuronal microtubules to promote stability and facilitate axonal transport. While neurons have the highest expression level of tau, oligodendrocytes and astrocytes also express endogenous tau, albeit at lower levels. Microglia do not express endogenous tau. In a pathological condition (*right*), tau becomes aberrantly aggregated in the form of various inclusions, impaired in its physiological functions, such as supporting microtubule stability. In neurons, tau can accumulate in the forms of NFTs, neuropil threads, or Pick bodies. Tau also accumulates in astrocytes, mostly in primary tauopathies such as PSP, CBD, and GGT, in the form of tufted astrocytes, astrocytic plaques, and GAIs. Moreover, tau aggregates in oligodendrocytes in the form of coiled bodies or GOIs. Microglia do not form tau inclusions, while accumulating studies have suggested that they may contribute to tau propagation

Aberrant neuronal and glial tau aggregation is a shared feature of tauopathies, but tau lesions appear in various forms across different cell types and within a given cell type depending upon the tauopathy (Fig. 3). Cell-type specificity, as well as morphological and structural heterogeneity, clearly indicates that distinct disease-specific pathomechanisms are implicated in tauopathies. While a growing body of literature continues to provide valuable insights on this aspect of tau pathophysiology, the underlying mechanisms remain unknown. It is still unclear why glial tau inclusions are frequent in primary tauopathies, but minimal in AD. Advanced proteomic approaches may help identify potential interactors or binders for tau in each tauopathy, or in different CNS cell types, that contribute to cell type-specific tau aggregation across diseases [190–192]. Moreover, it needs to be determined whether tau expressed in glia is the source of tau inclusions in astrocytes and oligodendroglia, or if the pathologic tau is derived from internalized neuronal tau. It has been suggested that neuronal tau is necessary to form tau aggregates in astrocytes, but not in oligodendrocytes [185], but further studies are needed to determine if astrocytes and oligodendrocytes develop tau lesions by distinct mechanisms.

In the recent years, the field has witnessed an exciting progress in development of various therapeutic strategies targeting tau, ranging from anti-aggregation agents to immunotherapy, with some of them being currently used in clinical trials [193–195]. It is unlikely that a therapeutic approach effective for one tauopathy will necessarily demonstrate similar efficacy for others. Advancing our understanding of what drives the cell-type specificity of tau aggregates in tauopathies will accelerate development of efficacious therapeutic strategies for each tauopathy. Moreover, it is intriguing that other pathological proteins, such as α-synuclein and TAR DNA binding protein 43 kDa (TDP-43), also aggregate in different cell types and have distinct anatomical distributions depending on the specific disease [196–198]. It has been suggested that distinct pathological properties of α-synuclein or TDP-43 are associated with their cell-type specificity [199, 200]. Elucidating mechanisms and related properties of tau aggregation in specific CNS cell types will greatly facilitate our understanding of a common pathogenic mechanism in neurodegenerative diseases.

## Data Availability

Not applicable.

## Abbreviations

Aβ: beta-amyloid
AD: Alzheimer’s disease
AGD: argyrophilic grain disease
APOE: apolipoprotein E
ARTAG: aging-related astrogliopathy
BBB: blood-brain barrier
C1q: complement component 1q
CBD: corticobasal degeneration
CNP: 2’,3’-cyclic nucleotide 3’-phosphodiesterase
CNS: central nervous system
cryo-EM: cryogenic electron microscopy
CTE: chronic traumatic encephalopathy
DEG: differentially expressed gene
EAAT2: excitatory amino acid transporter 2
FTD: frontotemporal dementia
FTDP-17: frontotemporal dementia and parkinsonism linked to chromosome 17
FTLD: frontotemporal lobar degeneration
GAI: globular astrocytic inclusion
GFAP: glial fibrillary acidic protein
GGT: globular glial tauopathy
GLT-1: glutamate transporter-1
GOI: globular oligodendroglial inclusion
GWAS: genome-wide association study
HSPG: heparan sulfate proteoglycan
LNT: Limbic-predominant Neuronal inclusion body 4R Tauopathy
MND: motor neuron disease
MOBP: myelin associated basic protein
NFT: neurofibrillary tangle
PART: primary age-related tauopathy
PDC: Parkinsonism-dementia complex
PHF: paired helical filament
PiD: Pick’s disease
PSP: :progressive supranuclear palsy
p-tau: hyperphosphorylated tau
PTM: post-translational modification
scRNA-seq: single-cell RNA sequencing
SF: straight filament
snRNA-seq: single-nucleus RNA sequencing
TFEB: transcription factor EB
TDP-43: TAR DNA binding protein 43 kDa
WT: wild-type

## References

[1] Wang Y, Mandelkow E (2016). Tau in physiology and pathology. Nat Rev Neurosci.

[2] Andreadis A, Brown WM, Kosik KS (1992). Structure and novel exons of the human tau gene. Biochemistry.

[3] Goedert M, Jakes R (1990). Expression of separate isoforms of human tau protein: correlation with the tau pattern in brain and effects on tubulin polymerization. EMBO J.

[4] Kosik KS, Orecchio LD, Bakalis S, Neve RL (1989). Developmentally regulated expression of specific tau sequences. Neuron.

[5] Binder LI, Frankfurter A, Rebhun LI (1985). The distribution of tau in the mammalian central nervous system. J Cell Biol.

[6] LoPresti P, Szuchet S, Papasozomenos SC, Zinkowski RP, Binder LI (1995). Functional implications for the microtubule-associated protein tau: localization in oligodendrocytes. Proc Natl Acad Sci U S A.

[7] Shin RW, Iwaki T, Kitamoto T, Tateishi J (1991). Hydrated autoclave pretreatment enhances tau immunoreactivity in formalin-fixed normal and Alzheimer’s disease brain tissues. Lab Invest.

[8] Simic G, Babic Leko M, Wray S, Harrington C, Delalle I, Jovanov-Milosevic N, Bazadona D, Buee L, de Silva R, Di Giovanni G (2016). Tau Protein Hyperphosphorylation and Aggregation in Alzheimer’s Disease and Other Tauopathies, and Possible Neuroprotective Strategies. Biomolecules.

[9] Cook C, Stankowski JN, Carlomagno Y, Stetler C, Petrucelli L (2014). Acetylation: a new key to unlock tau’s role in neurodegeneration. Alzheimers Res Ther.

[10] Ciechanover A, Kwon YT (2015). Degradation of misfolded proteins in neurodegenerative diseases: therapeutic targets and strategies. Exp Mol Med.

[11] Quinn JP, Corbett NJ, Kellett KAB, Hooper NM (2018). Tau Proteolysis in the Pathogenesis of Tauopathies: Neurotoxic Fragments and Novel Biomarkers. J Alzheimers Dis.

[12] Kuret J, Chirita CN, Congdon EE, Kannanayakal T, Li G, Necula M, Yin H, Zhong Q (2005). Pathways of tau fibrillization. Biochim Biophys Acta.

[13] Cowan CM, Mudher A (2013). Are tau aggregates toxic or protective in tauopathies?. Front Neurol.

[14] Kidd M (1963). Paired helical filaments in electron microscopy of Alzheimer’s disease. Nature.

[15] Grundke-Iqbal I, Iqbal K, Tung YC, Quinlan M, Wisniewski HM, Binder LI (1986). Abnormal phosphorylation of the microtubule-associated protein tau (tau) in Alzheimer cytoskeletal pathology. Proc Natl Acad Sci U S A.

[16] Morsch R, Simon W, Coleman PD (1999). Neurons may live for decades with neurofibrillary tangles. J Neuropathol Exp Neurol.

[17] Spires-Jones TL, Stoothoff WH, de Calignon A, Jones PB, Hyman BT (2009). Tau pathophysiology in neurodegeneration: a tangled issue. Trends Neurosci.

[18] Gibbons GS, Lee VMY, Trojanowski JQ (2019). Mechanisms of Cell-to-Cell Transmission of Pathological Tau: A Review. JAMA Neurol.

[19] Iba M, Guo JL, McBride JD, Zhang B, Trojanowski JQ, Lee VM (2013). Synthetic tau fibrils mediate transmission of neurofibrillary tangles in a transgenic mouse model of Alzheimer’s-like tauopathy. J Neurosci.

[20] Peeraer E, Bottelbergs A, Van Kolen K, Stancu IC, Vasconcelos B, Mahieu M, Duytschaever H, Ver Donck L, Torremans A, Sluydts E (2015). Intracerebral injection of preformed synthetic tau fibrils initiates widespread tauopathy and neuronal loss in the brains of tau transgenic mice. Neurobiol Dis.

[21] Sanders DW, Kaufman SK, DeVos SL, Sharma AM, Mirbaha H, Li A, Barker SJ, Foley AC, Thorpe JR, Serpell LC (2014). Distinct tau prion strains propagate in cells and mice and define different tauopathies. Neuron.

[22] Kaufman SK, Sanders DW, Thomas TL, Ruchinskas AJ, Vaquer-Alicea J, Sharma AM, Miller TM, Diamond MI (2016). Tau Prion Strains Dictate Patterns of Cell Pathology, Progression Rate, and Regional Vulnerability In Vivo. Neuron.

[23] Clavaguera F, Bolmont T, Crowther RA, Abramowski D, Frank S, Probst A, Fraser G, Stalder AK, Beibel M, Staufenbiel M (2009). Transmission and spreading of tauopathy in transgenic mouse brain. Nat Cell Biol.

[24] Ahmed Z, Cooper J, Murray TK, Garn K, McNaughton E, Clarke H, Parhizkar S, Ward MA, Cavallini A, Jackson S (2014). A novel in vivo model of tau propagation with rapid and progressive neurofibrillary tangle pathology: the pattern of spread is determined by connectivity, not proximity. Acta Neuropathol.

[25] Lasagna-Reeves CA, Castillo-Carranza DL, Sengupta U, Sarmiento J, Troncoso J, Jackson GR, Kayed R (2012). Identification of oligomers at early stages of tau aggregation in Alzheimer’s disease. FASEB J.

[26] Clavaguera F, Akatsu H, Fraser G, Crowther RA, Frank S, Hench J, Probst A, Winkler DT, Reichwald J, Staufenbiel M (2013). Brain homogenates from human tauopathies induce tau inclusions in mouse brain. Proc Natl Acad Sci U S A.

[27] Boluda S, Iba M, Zhang B, Raible KM, Lee VM, Trojanowski JQ (2015). Differential induction and spread of tau pathology in young PS19 tau transgenic mice following intracerebral injections of pathological tau from Alzheimer’s disease or corticobasal degeneration brains. Acta Neuropathol.

[28] Narasimhan S, Guo JL, Changolkar L, Stieber A, McBride JD, Silva LV, He Z, Zhang B, Gathagan RJ, Trojanowski JQ, Lee VMY (2017). Pathological Tau Strains from Human Brains Recapitulate the Diversity of Tauopathies in Nontransgenic Mouse Brain. J Neurosci.

[29] Chung DC, Carlomagno Y, Cook CN, Jansen-West K, Daughrity L, Lewis-Tuffin LJ, Castanedes-Casey M, DeTure M, Dickson DW, Petrucelli L (2019). Tau exhibits unique seeding properties in globular glial tauopathy. Acta Neuropathol Commun.

[30] Hardy J (2006). Alzheimer’s disease: the amyloid cascade hypothesis: an update and reappraisal. J Alzheimers Dis.

[31] Crapper McLachlarf DR, McLachlan CD, Krishnan B, Krishnan SS, Dalton AJ, Steele JC (1989). Aluminium and calcium in soil and food from Guam, Palau and Jamaica: Implications for amyotrophic lateral sclerosis and parkinsonism-dementia syndromes of Guam. Environ Geochem Health.

[32] McGeer PL, Steele JC (2011). The ALS/PDC syndrome of Guam: potential biomarkers for an enigmatic disorder. Prog Neurobiol.

[33] Mackenzie IR, Neumann M (2016). Molecular neuropathology of frontotemporal dementia: insights into disease mechanisms from postmortem studies. J Neurochem.

[34] Neumann M, Schulz-Schaeffer W, Crowther RA, Smith MJ, Spillantini MG, Goedert M, Kretzschmar HA (2001). Pick’s disease associated with the novel Tau gene mutation K369I. Ann Neurol.

[35] Tacik P, DeTure M, Hinkle KM, Lin WL, Sanchez-Contreras M, Carlomagno Y, Pedraza O, Rademakers R, Ross OA, Wszolek ZK, Dickson DW (2015). A Novel Tau Mutation in Exon 12, p.Q336H, Causes Hereditary Pick Disease. J Neuropathol Exp Neurol.

[36] Dickson DW (1998). Pick’s disease: a modern approach. Brain Pathol.

[37] Kovacs GG, Rozemuller AJ, van Swieten JC, Gelpi E, Majtenyi K, Al-Sarraj S, Troakes C, Bodi I, King A, Hortobagyi T (2013). Neuropathology of the hippocampus in FTLD-Tau with Pick bodies: a study of the BrainNet Europe Consortium. Neuropathol Appl Neurobiol.

[38] Dickson DW, Yen SH, Horoupian DS (1986). Pick body-like inclusions in the dentate fascia of the hippocampus in Alzheimer’s disease. Acta Neuropathol.

[39] Tacik P, DeTure MA, Carlomagno Y, Lin WL, Murray ME, Baker MC, Josephs KA, Boeve BF, Wszolek ZK, Graff-Radford NR (2017). FTDP-17 with Pick body-like inclusions associated with a novel tau mutation, p.E372G. Brain Pathol.

[40] Komori T (1999). Tau-positive glial inclusions in progressive supranuclear palsy, corticobasal degeneration and Pick’s disease. Brain Pathol.

[41] Sakae N, Roemer SF, Bieniek KF, Murray ME, Baker MC, Kasanuki K, Graff-Radford NR, Petrucelli L, Van Blitterswijk M, Rademakers R, Dickson DW (2019). Microglia in frontotemporal lobar degeneration with progranulin or C9ORF72 mutations. Ann Clin Transl Neurol.

[42] Fujioka S, Algom AA, Murray ME, Strongosky A, Soto-Ortolaza AI, Rademakers R, Ross OA, Wszolek ZK, Dickson DW (2013). Similarities between familial and sporadic autopsy-proven progressive supranuclear palsy. Neurology.

[43] Williams DR, de Silva R, Paviour DC, Pittman A, Watt HC, Kilford L, Holton JL, Revesz T, Lees AJ (2005). Characteristics of two distinct clinical phenotypes in pathologically proven progressive supranuclear palsy: Richardson’s syndrome and PSP-parkinsonism. Brain.

[44] Hoglinger GU, Respondek G, Stamelou M, Kurz C, Josephs KA, Lang AE, Mollenhauer B, Muller U, Nilsson C, Whitwell JL (2017). Clinical diagnosis of progressive supranuclear palsy: The movement disorder society criteria. Mov Disord.

[45] Rohrer JD, Paviour D, Vandrovcova J, Hodges J, de Silva R, Rossor MN (2011). Novel L284R MAPT mutation in a family with an autosomal dominant progressive supranuclear palsy syndrome. Neurodegener Dis.

[46] Fujioka S, Sanchez Contreras MY, Strongosky AJ, Ogaki K, Whaley NR, Tacik PM, van Gerpen JA, Uitti RJ, Ross OA, Wszolek ZK (2015). Three sib-pairs of autopsy-confirmed progressive supranuclear palsy. Parkinsonism Relat Disord.

[47] Im SY, Kim YE, Kim YJ (2015). Genetics of Progressive Supranuclear Palsy. J Mov Disord.

[48] Liu F, Gong CX (2008). Tau exon 10 alternative splicing and tauopathies. Mol Neurodegener.

[49] Stefansson H, Helgason A, Thorleifsson G, Steinthorsdottir V, Masson G, Barnard J, Baker A, Jonasdottir A, Ingason A, Gudnadottir VG (2005). A common inversion under selection in Europeans. Nat Genet.

[50] Hoglinger GU, Melhem NM, Dickson DW, Sleiman PM, Wang LS, Klei L, Rademakers R, de Silva R, Litvan I, Riley DE (2011). Identification of common variants influencing risk of the tauopathy progressive supranuclear palsy. Nat Genet.

[51] Zhao N, Liu CC, Van Ingelgom AJ, Linares C, Kurti A, Knight JA, Heckman MG, Diehl NN, Shinohara M, Martens YA (2018). APOE epsilon2 is associated with increased tau pathology in primary tauopathy. Nat Commun.

[52] Dickson DW, Rademakers R, Hutton ML (2007). Progressive supranuclear palsy: pathology and genetics. Brain Pathol.

[53] Song YJ, Halliday GM, Holton JL, Lashley T, O’Sullivan SS, McCann H, Lees AJ, Ozawa T, Williams DR, Lockhart PJ, Revesz TR (2009). Degeneration in different parkinsonian syndromes relates to astrocyte type and astrocyte protein expression. J Neuropathol Exp Neurol.

[54] Togo T, Dickson DW (2002). Tau accumulation in astrocytes in progressive supranuclear palsy is a degenerative rather than a reactive process. Acta Neuropathol.

[55] Rossi G, Marelli C, Farina L, Laura M, Maria Basile A, Ciano C, Tagliavini F, Pareyson D (2008). The G389R mutation in the MAPT gene presenting as sporadic corticobasal syndrome. Mov Disord.

[56] Kouri N, Carlomagno Y, Baker M, Liesinger AM, Caselli RJ, Wszolek ZK, Petrucelli L, Boeve BF, Parisi JE, Josephs KA (2014). Novel mutation in MAPT exon 13 (p.N410H) causes corticobasal degeneration. Acta Neuropathol.

[57] Houlden H, Baker M, Morris HR, MacDonald N, Pickering-Brown S, Adamson J, Lees AJ, Rossor MN, Quinn NP, Kertesz A (2001). Corticobasal degeneration and progressive supranuclear palsy share a common tau haplotype. Neurology.

[58] Armstrong MJ, Litvan I, Lang AE, Bak TH, Bhatia KP, Borroni B, Boxer AL, Dickson DW, Grossman M, Hallett M (2013). Criteria for the diagnosis of corticobasal degeneration. Neurology.

[59] Dickson DW (1999). Neuropathologic differentiation of progressive supranuclear palsy and corticobasal degeneration. J Neurol.

[60] Dickson DW, Yen SH, Suzuki KI, Davies P, Garcia JH, Hirano A (1986). Ballooned neurons in select neurodegenerative diseases contain phosphorylated neurofilament epitopes. Acta Neuropathol.

[61] Lowe J, Errington DR, Lennox G, Pike I, Spendlove I, Landon M, Mayer RJ (1992). Ballooned neurons in several neurodegenerative diseases and stroke contain alpha B crystallin. Neuropathol Appl Neurobiol.

[62] Feany MB, Dickson DW (1995). Widespread cytoskeletal pathology characterizes corticobasal degeneration. Am J Pathol.

[63] Dickson DW, Feany MB, Yen SH, Mattiace LA, Davies P (1996). Cytoskeletal pathology in non-Alzheimer degenerative dementia: new lesions in diffuse Lewy body disease, Pick’s disease, and corticobasal degeneration. J Neural Transm Suppl.

[64] Komori T, Arai N, Oda M, Nakayama H, Mori H, Yagishita S, Takahashi T, Amano N, Murayama S, Murakami S (1998). Astrocytic plaques and tufts of abnormal fibers do not coexist in corticobasal degeneration and progressive supranuclear palsy. Acta Neuropathol.

[65] Braak H, Braak E (1991). Neuropathological stageing of Alzheimer-related changes. Acta Neuropathol.

[66] Zarranz JJ, Ferrer I, Lezcano E, Forcadas MI, Eizaguirre B, Atares B, Puig B, Gomez-Esteban JC, Fernandez-Maiztegui C, Rouco I (2005). A novel mutation (K317M) in the MAPT gene causes FTDP and motor neuron disease. Neurology.

[67] Tacik P, DeTure M, Lin WL, Sanchez Contreras M, Wojtas A, Hinkle KM, Fujioka S, Baker MC, Walton RL, Carlomagno Y (2015). A novel tau mutation, p.K317N, causes globular glial tauopathy. Acta Neuropathol.

[68] Tacik P, Sanchez-Contreras M, DeTure M, Murray ME, Rademakers R, Ross OA, Wszolek ZK, Parisi JE, Knopman DS, Petersen RC, Dickson DW (2017). Clinicopathologic heterogeneity in frontotemporal dementia and parkinsonism linked to chromosome 17 (FTDP-17) due to microtubule-associated protein tau (MAPT) p.P301L mutation, including a patient with globular glial tauopathy. Neuropathol Appl Neurobiol.

[69] Josephs KA, Katsuse O, Beccano-Kelly DA, Lin WL, Uitti RJ, Fujino Y, Boeve BF, Hutton ML, Baker MC, Dickson DW (2006). Atypical progressive supranuclear palsy with corticospinal tract degeneration. J Neuropathol Exp Neurol.

[70] Bigio EH, Lipton AM, Yen SH, Hutton ML, Baker M, Nacharaju P, White CL 3rd, Davies P, Lin W, Dickson DW. Frontal lobe dementia with novel tauopathy: sporadic multiple system tauopathy with dementia. J Neuropathol Exp Neurol. 2001;60:328–41. 10.1093/jnen/60.4.328 11305868

[71] Ahmed Z, Bigio EH, Budka H, Dickson DW, Ferrer I, Ghetti B, Giaccone G, Hatanpaa KJ, Holton JL, Josephs KA (2013). Globular glial tauopathies (GGT): consensus recommendations. Acta Neuropathol.

[72] Saijo E, Metrick MA 2nd, Koga S, Parchi P, Litvan I, Spina S, Boxer A, Rojas JC, Galasko D, Kraus A, et al. 4-Repeat tau seeds and templating subtypes as brain and CSF biomarkers of frontotemporal lobar degeneration. Acta Neuropathol. 2020;139:63–77. 10.1007/s00401-019-02080-2 PMC7192393 31616982

[73] Ferrer I, Andres-Benito P, Zelaya MV, Aguirre MEE, Carmona M, Ausin K, Lachen-Montes M, Fernandez-Irigoyen J, Santamaria E, Del Rio JA (2020). Familial globular glial tauopathy linked to MAPT mutations: molecular neuropathology and seeding capacity of a prototypical mixed neuronal and glial tauopathy. Acta Neuropathol.

[74] Braak H, Braak E (1987). Argyrophilic grains: characteristic pathology of cerebral cortex in cases of adult onset dementia without Alzheimer changes. Neurosci Lett.

[75] Togo T, Sahara N, Yen SH, Cookson N, Ishizawa T, Hutton M, de Silva R, Lees A, Dickson DW (2002). Argyrophilic grain disease is a sporadic 4-repeat tauopathy. J Neuropathol Exp Neurol.

[76] Togo T, Cookson N, Dickson DW (2002). Argyrophilic grain disease: neuropathology, frequency in a dementia brain bank and lack of relationship with apolipoprotein E. Brain Pathol.

[77] Saito Y, Ruberu NN, Sawabe M, Arai T, Tanaka N, Kakuta Y, Yamanouchi H, Murayama S (2004). Staging of argyrophilic grains: an age-associated tauopathy. J Neuropathol Exp Neurol.

[78] Rodriguez RD, Grinberg LT (2015). Argyrophilic grain disease: An underestimated tauopathy. Dement Neuropsychol.

[79] Bancher C, Jellinger KA (1994). Neurofibrillary tangle predominant form of senile dementia of Alzheimer type: a rare subtype in very old subjects. Acta Neuropathol.

[80] Crary JF, Trojanowski JQ, Schneider JA, Abisambra JF, Abner EL, Alafuzoff I, Arnold SE, Attems J, Beach TG, Bigio EH (2014). Primary age-related tauopathy (PART): a common pathology associated with human aging. Acta Neuropathol.

[81] de Silva R, Lashley T, Strand C, Shiarli AM, Shi J, Tian J, Bailey KL, Davies P, Bigio EH, Arima K (2006). An immunohistochemical study of cases of sporadic and inherited frontotemporal lobar degeneration using 3R- and 4R-specific tau monoclonal antibodies. Acta Neuropathol.

[82] Besser LM, Crary JF, Mock C, Kukull WA (2017). Comparison of symptomatic and asymptomatic persons with primary age-related tauopathy. Neurology.

[83] Jellinger KA (2018). Different patterns of hippocampal tau pathology in Alzheimer’s disease and PART. Acta Neuropathol.

[84] Kovacs GG, Ferrer I, Grinberg LT, Alafuzoff I, Attems J, Budka H, Cairns NJ, Crary JF, Duyckaerts C, Ghetti B (2016). Aging-related tau astrogliopathy (ARTAG): harmonized evaluation strategy. Acta Neuropathol.

[85] Kovacs GG, Xie SX, Robinson JL, Lee EB, Smith DH, Schuck T, Lee VM, Trojanowski JQ (2018). Sequential stages and distribution patterns of aging-related tau astrogliopathy (ARTAG) in the human brain. Acta Neuropathol Commun.

[86] Kovacs GG, Yousef A, Kaindl S, Lee VM, Trojanowski JQ (2018). Connexin-43 and aquaporin-4 are markers of ageing-related tau astrogliopathy (ARTAG)-related astroglial response. Neuropathol Appl Neurobiol.

[87] DeTure MA, Dickson DW (2019). The neuropathological diagnosis of Alzheimer’s disease. Mol Neurodegener.

[88] Braak H, Alafuzoff I, Arzberger T, Kretzschmar H, Del Tredici K (2006). Staging of Alzheimer disease-associated neurofibrillary pathology using paraffin sections and immunocytochemistry. Acta Neuropathol.

[89] Bancher C, Brunner C, Lassmann H, Budka H, Jellinger K, Wiche G, Seitelberger F, Grundke-Iqbal I, Iqbal K, Wisniewski HM (1989). Accumulation of abnormally phosphorylated tau precedes the formation of neurofibrillary tangles in Alzheimer’s disease. Brain Res.

[90] McKee AC, Cantu RC, Nowinski CJ, Hedley-Whyte ET, Gavett BE, Budson AE, Santini VE, Lee HS, Kubilus CA, Stern RA (2009). Chronic traumatic encephalopathy in athletes: progressive tauopathy after repetitive head injury. J Neuropathol Exp Neurol.

[91] Goldstein LE, Fisher AM, Tagge CA, Zhang XL, Velisek L, Sullivan JA, Upreti C, Kracht JM, Ericsson M, Wojnarowicz MW (2012). Chronic traumatic encephalopathy in blast-exposed military veterans and a blast neurotrauma mouse model. Sci Transl Med.

[92] Bieniek KF, Blessing MM, Heckman MG, Diehl NN, Serie AM, Paolini MA 2nd, Boeve BF, Savica R, Reichard RR, Dickson DW. Association between contact sports participation and chronic traumatic encephalopathy: a retrospective cohort study. Brain Pathol. 2020;30:63–74. 10.1111/bpa.12757 PMC6916416 31199537

[93] McKee AC, Gavett BE, Stern RA, Nowinski CJ, Cantu RC, Kowall NW, Perl DP, Hedley-Whyte ET, Price B, Sullivan C (2010). TDP-43 proteinopathy and motor neuron disease in chronic traumatic encephalopathy. J Neuropathol Exp Neurol.

[94] McKee AC, Stern RA, Nowinski CJ, Stein TD, Alvarez VE, Daneshvar DH, Lee HS, Wojtowicz SM, Hall G, Baugh CM (2013). The spectrum of disease in chronic traumatic encephalopathy. Brain.

[95] Bieniek KF, Ross OA, Cormier KA, Walton RL, Soto-Ortolaza A, Johnston AE, DeSaro P, Boylan KB, Graff-Radford NR, Wszolek ZK (2015). Chronic traumatic encephalopathy pathology in a neurodegenerative disorders brain bank. Acta Neuropathol.

[96] McKee AC, Stein TD, Kiernan PT, Alvarez VE (2015). The neuropathology of chronic traumatic encephalopathy. Brain Pathol.

[97] Bieniek KF, Cairns NJ, Crary JF, Dickson DW, Folkerth RD, Keene CD, Litvan I, Perl DP, Stein TD, Vonsattel JP (2021). The Second NINDS/NIBIB Consensus Meeting to Define Neuropathological Criteria for the Diagnosis of Chronic Traumatic Encephalopathy. J Neuropathol Exp Neurol.

[98] Winton MJ, Joyce S, Zhukareva V, Practico D, Perl DP, Galasko D, Craig U, Trojanowski JQ, Lee VM (2006). Characterization of tau pathologies in gray and white matter of Guam parkinsonism-dementia complex. Acta Neuropathol.

[99] Ince PG, Codd GA (2005). Return of the cycad hypothesis - does the amyotrophic lateral sclerosis/parkinsonism dementia complex (ALS/PDC) of Guam have new implications for global health?. Neuropathol Appl Neurobiol.

[100] Caparros-Lefebvre D, Elbaz A (1999). Possible relation of atypical parkinsonism in the French West Indies with consumption of tropical plants: a case-control study. Caribbean Parkinsonism Study Group. Lancet.

[101] Caparros-Lefebvre D, Sergeant N, Lees A, Camuzat A, Daniel S, Lannuzel A, Brice A, Tolosa E, Delacourte A, Duyckaerts C (2002). Guadeloupean parkinsonism: a cluster of progressive supranuclear palsy-like tauopathy. Brain.

[102] Caparros-Lefebvre D, Golbe LI, Deramecourt V, Maurage CA, Huin V, Buee-Scherrer V, Obriot H, Sablonniere B, Caparros F, Buee L, Lees AJ (2015). A geographical cluster of progressive supranuclear palsy in northern France. Neurology.

[103] Drechsel DN, Hyman AA, Cobb MH, Kirschner MW (1992). Modulation of the dynamic instability of tubulin assembly by the microtubule-associated protein tau. Mol Biol Cell.

[104] Vershinin M, Carter BC, Razafsky DS, King SJ, Gross SP (2007). Multiple-motor based transport and its regulation by Tau. Proc Natl Acad Sci U S A.

[105] Dixit R, Ross JL, Goldman YE, Holzbaur EL (2008). Differential regulation of dynein and kinesin motor proteins by tau. Science.

[106] Caceres A, Kosik KS (1990). Inhibition of neurite polarity by tau antisense oligonucleotides in primary cerebellar neurons. Nature.

[107] Knops J, Kosik KS, Lee G, Pardee JD, Cohen-Gould L, McConlogue L (1991). Overexpression of tau in a nonneuronal cell induces long cellular processes. J Cell Biol.

[108] Leyns CEG, Holtzman DM (2017). Glial contributions to neurodegeneration in tauopathies. Mol Neurodegener.

[109] Pekny M, Pekna M (2014). Astrocyte reactivity and reactive astrogliosis: costs and benefits. Physiol Rev.

[110] Ferrer I (2017). Diversity of astroglial responses across human neurodegenerative disorders and brain aging. Brain Pathol.

[111] Perez-Nievas BG, Serrano-Pozo A (2018). Deciphering the Astrocyte Reaction in Alzheimer’s Disease. Front Aging Neurosci.

[112] Liddelow SA, Guttenplan KA, Clarke LE, Bennett FC, Bohlen CJ, Schirmer L, Bennett ML, Munch AE, Chung WS, Peterson TC (2017). Neurotoxic reactive astrocytes are induced by activated microglia. Nature.

[113] Joshi AU, Minhas PS, Liddelow SA, Haileselassie B, Andreasson KI, Dorn GW 2nd, Mochly-Rosen D. Fragmented mitochondria released from microglia trigger A1 astrocytic response and propagate inflammatory neurodegeneration. Nat Neurosci. 2019;22:1635–48. 10.1038/s41593-019-0486-0 PMC6764589 31551592

[114] Ferrer I, Andres-Benito P, Zelaya MV, Aguirre MEE, Carmona M, Ausin K, Lachen-Montes M, Fernandez-Irigoyen J, Santamaria E, Del Rio JA. Familial globular glial tauopathy linked to MAPT mutations: molecular neuropathology and seeding capacity of a prototypical mixed neuronal and glial tauopathy. Acta Neuropathol. 2020. 10.1007/s00401-019-02122-9 PMC7096369 31907603

[115] Dabir DV, Robinson MB, Swanson E, Zhang B, Trojanowski JQ, Lee VM, Forman MS (2006). Impaired glutamate transport in a mouse model of tau pathology in astrocytes. J Neurosci.

[116] Galloway DA, Phillips AEM, Owen DRJ, Moore CS (2019). Phagocytosis in the Brain: Homeostasis and Disease. Front Immunol.

[117] Ries M, Sastre M (2016). Mechanisms of Abeta Clearance and Degradation by Glial Cells. Front Aging Neurosci.

[118] Martini-Stoica H, Cole AL, Swartzlander DB, Chen F, Wan YW, Bajaj L, Bader DA, Lee VMY, Trojanowski JQ, Liu Z (2018). TFEB enhances astroglial uptake of extracellular tau species and reduces tau spreading. J Exp Med.

[119] Perea JR, Lopez E, Diez-Ballesteros JC, Avila J, Hernandez F, Bolos M (2019). Extracellular Monomeric Tau Is Internalized by Astrocytes. Front Neurosci.

[120] Sollvander S, Nikitidou E, Brolin R, Soderberg L, Sehlin D, Lannfelt L, Erlandsson A (2016). Accumulation of amyloid-beta by astrocytes result in enlarged endosomes and microvesicle-induced apoptosis of neurons. Mol Neurodegener.

[121] Garwood CJ, Pooler AM, Atherton J, Hanger DP, Noble W (2011). Astrocytes are important mediators of Abeta-induced neurotoxicity and tau phosphorylation in primary culture. Cell Death Dis.

[122] Kovacs GG, Lukic MJ, Irwin DJ, Arzberger T, Respondek G, Lee EB, Coughlin D, Giese A, Grossman M, Kurz C (2020). Distribution patterns of tau pathology in progressive supranuclear palsy. Acta Neuropathol.

[123] Klein C, Kramer EM, Cardine AM, Schraven B, Brandt R, Trotter J (2002). Process outgrowth of oligodendrocytes is promoted by interaction of fyn kinase with the cytoskeletal protein tau. J Neurosci.

[124] LoPresti P (2002). Regulation and differential expression of tau mRNA isoforms as oligodendrocytes mature in vivo: implications for myelination. Glia.

[125] Seiberlich V, Bauer NG, Schwarz L, Ffrench-Constant C, Goldbaum O, Richter-Landsberg C (2015). Downregulation of the microtubule associated protein tau impairs process outgrowth and myelin basic protein mRNA transport in oligodendrocytes. Glia.

[126] LoPresti P. Tau in Oligodendrocytes Takes Neurons in Sickness and in Health. Int J Mol Sci. 2018;19. 10.3390/ijms19082408 PMC6121290 30111714

[127] Nasrabady SE, Rizvi B, Goldman JE, Brickman AM (2018). White matter changes in Alzheimer’s disease: a focus on myelin and oligodendrocytes. Acta Neuropathol Commun.

[128] Scheltens P, Barkhof F, Valk J, Algra PR, van der Hoop RG, Nauta J, Wolters EC (1992). White matter lesions on magnetic resonance imaging in clinically diagnosed Alzheimer’s disease. Evidence for heterogeneity. Brain.

[129] Whitwell JL, Master AV, Avula R, Kantarci K, Eggers SD, Edmonson HA, Jack CR, Josephs KA (2011). Clinical correlates of white matter tract degeneration in progressive supranuclear palsy. Arch Neurol.

[130] Forman MS, Zhukareva V, Bergeron C, Chin SS, Grossman M, Clark C, Lee VM, Trojanowski JQ (2002). Signature tau neuropathology in gray and white matter of corticobasal degeneration. Am J Pathol.

[131] Kovacs GG (2015). Invited review: Neuropathology of tauopathies: principles and practice. Neuropathol Appl Neurobiol.

[132] Ferrer I, Aguilo Garcia M, Carmona M, Andres-Benito P, Torrejon-Escribano B, Garcia-Esparcia P, Del Rio JA (2019). Involvement of Oligodendrocytes in Tau Seeding and Spreading in Tauopathies. Front Aging Neurosci.

[133] Colonna M, Butovsky O (2017). Microglia Function in the Central Nervous System During Health and Neurodegeneration. Annu Rev Immunol.

[134] Nimmerjahn A, Kirchhoff F, Helmchen F (2005). Resting microglial cells are highly dynamic surveillants of brain parenchyma in vivo. Science.

[135] Harold D, Abraham R, Hollingworth P, Sims R, Gerrish A, Hamshere ML, Pahwa JS, Moskvina V, Dowzell K, Williams A (2009). Genome-wide association study identifies variants at CLU and PICALM associated with Alzheimer’s disease. Nat Genet.

[136] Lambert JC, Heath S, Even G, Campion D, Sleegers K, Hiltunen M, Combarros O, Zelenika D, Bullido MJ, Tavernier B (2009). Genome-wide association study identifies variants at CLU and CR1 associated with Alzheimer’s disease. Nat Genet.

[137] Jun G, Naj AC, Beecham GW, Wang LS, Buros J, Gallins PJ, Buxbaum JD, Ertekin-Taner N, Fallin MD, Friedland R (2010). Meta-analysis confirms CR1, CLU, and PICALM as alzheimer disease risk loci and reveals interactions with APOE genotypes. Arch Neurol.

[138] Lambert JC, Zelenika D, Hiltunen M, Chouraki V, Combarros O, Bullido MJ, Tognoni G, Fievet N, Boland A, Arosio B (2011). Evidence of the association of BIN1 and PICALM with the AD risk in contrasting European populations. Neurobiol Aging.

[139] Hollingworth P, Harold D, Sims R, Gerrish A, Lambert JC, Carrasquillo MM, Abraham R, Hamshere ML, Pahwa JS, Moskvina V (2011). Common variants at ABCA7, MS4A6A/MS4A4E, EPHA1, CD33 and CD2AP are associated with Alzheimer’s disease. Nat Genet.

[140] Naj AC, Jun G, Beecham GW, Wang LS, Vardarajan BN, Buros J, Gallins PJ, Buxbaum JD, Jarvik GP, Crane PK (2011). Common variants at MS4A4/MS4A6E, CD2AP, CD33 and EPHA1 are associated with late-onset Alzheimer’s disease. Nat Genet.

[141] Lambert JC, Ibrahim-Verbaas CA, Harold D, Naj AC, Sims R, Bellenguez C, DeStafano AL, Bis JC, Beecham GW, Grenier-Boley B (2013). Meta-analysis of 74,046 individuals identifies 11 new susceptibility loci for Alzheimer’s disease. Nat Genet.

[142] Jansen IE, Savage JE, Watanabe K, Bryois J, Williams DM, Steinberg S, Sealock J, Karlsson IK, Hagg S, Athanasiu L (2019). Genome-wide meta-analysis identifies new loci and functional pathways influencing Alzheimer’s disease risk. Nat Genet.

[143] Kunkle BW, Grenier-Boley B, Sims R, Bis JC, Damotte V, Naj AC, Boland A, Vronskaya M, van der Lee SJ, Amlie-Wolf A (2019). Genetic meta-analysis of diagnosed Alzheimer’s disease identifies new risk loci and implicates Abeta, tau, immunity and lipid processing. Nat Genet.

[144] Ishizawa K, Dickson DW (2001). Microglial activation parallels system degeneration in progressive supranuclear palsy and corticobasal degeneration. J Neuropathol Exp Neurol.

[145] Odawara T, Iseki E, Kosaka K, Akiyama H, Ikeda K, Yamamoto T (1995). Investigation of tau-2 positive microglia-like cells in the subcortical nuclei of human neurodegenerative disorders. Neurosci Lett.

[146] Ghoshal N, Garcia-Sierra F, Fu Y, Beckett LA, Mufson EJ, Kuret J, Berry RW, Binder LI (2001). Tau-66: evidence for a novel tau conformation in Alzheimer’s disease. J Neurochem.

[147] Bolos M, Llorens-Martin M, Jurado-Arjona J, Hernandez F, Rabano A, Avila J (2016). Direct Evidence of Internalization of Tau by Microglia In Vitro and In Vivo. J Alzheimers Dis.

[148] Asai H, Ikezu S, Tsunoda S, Medalla M, Luebke J, Haydar T, Wolozin B, Butovsky O, Kugler S, Ikezu T (2015). Depletion of microglia and inhibition of exosome synthesis halt tau propagation. Nat Neurosci.

[149] Clayton K, Delpech JC, Herron S, Iwahara N, Ericsson M, Saito T, Saido TC, Ikezu S, Ikezu T (2021). Plaque associated microglia hyper-secrete extracellular vesicles and accelerate tau propagation in a humanized APP mouse model. Mol Neurodegener.

[150] Behrendt A, Bichmann M, Ercan-Herbst E, Haberkant P, Schondorf DC, Wolf M, Fahim SA, Murolo E, Ehrnhoefer DE (2019). Asparagine endopeptidase cleaves tau at N167 after uptake into microglia. Neurobiol Dis.

[151] Allen M, Wang X, Serie DJ, Strickland SL, Burgess JD, Koga S, Younkin CS, Nguyen TT, Malphrus KG, Lincoln SJ (2018). Divergent brain gene expression patterns associate with distinct cell-specific tau neuropathology traits in progressive supranuclear palsy. Acta Neuropathol.

[152] Macosko EZ, Basu A, Satija R, Nemesh J, Shekhar K, Goldman M, Tirosh I, Bialas AR, Kamitaki N, Martersteck EM (2015). Highly Parallel Genome-wide Expression Profiling of Individual Cells Using Nanoliter Droplets. Cell.

[153] Lake BB, Ai R, Kaeser GE, Salathia NS, Yung YC, Liu R, Wildberg A, Gao D, Fung HL, Chen S (2016). Neuronal subtypes and diversity revealed by single-nucleus RNA sequencing of the human brain. Science.

[154] Habib N, Avraham-Davidi I, Basu A, Burks T, Shekhar K, Hofree M, Choudhury SR, Aguet F, Gelfand E, Ardlie K (2017). Massively parallel single-nucleus RNA-seq with DroNc-sEq. Nat Methods.

[155] Lake BB, Chen S, Sos BC, Fan J, Kaeser GE, Yung YC, Duong TE, Gao D, Chun J, Kharchenko PV, Zhang K (2018). Integrative single-cell analysis of transcriptional and epigenetic states in the human adult brain. Nat Biotechnol.

[156] Fu H, Possenti A, Freer R, Nakano Y, Hernandez Villegas NC, Tang M, Cauhy PVM, Lassus BA, Chen S, Fowler SL (2019). A tau homeostasis signature is linked with the cellular and regional vulnerability of excitatory neurons to tau pathology. Nat Neurosci.

[157] Mathys H, Davila-Velderrain J, Peng Z, Gao F, Mohammadi S, Young JZ, Menon M, He L, Abdurrob F, Jiang X (2019). Single-cell transcriptomic analysis of Alzheimer’s disease. Nature.

[158] Del-Aguila JL, Li Z, Dube U, Mihindukulasuriya KA, Budde JP, Fernandez MV, Ibanez L, Bradley J, Wang F, Bergmann K (2019). A single-nuclei RNA sequencing study of Mendelian and sporadic AD in the human brain. Alzheimers Res Ther.

[159] Grubman A, Chew G, Ouyang JF, Sun G, Choo XY, McLean C, Simmons RK, Buckberry S, Vargas-Landin DB, Poppe D (2019). A single-cell atlas of entorhinal cortex from individuals with Alzheimer’s disease reveals cell-type-specific gene expression regulation. Nat Neurosci.

[160] Murata K, Wolf M (2018). Cryo-electron microscopy for structural analysis of dynamic biological macromolecules. Biochim Biophys Acta Gen Subj.

[161] Fitzpatrick AWP, Falcon B, He S, Murzin AG, Murshudov G, Garringer HJ, Crowther RA, Ghetti B, Goedert M, Scheres SHW (2017). Cryo-EM structures of tau filaments from Alzheimer’s disease. Nature.

[162] Falcon B, Zhang W, Schweighauser M, Murzin AG, Vidal R, Garringer HJ, Ghetti B, Scheres SHW, Goedert M (2018). Tau filaments from multiple cases of sporadic and inherited Alzheimer’s disease adopt a common fold. Acta Neuropathol.

[163] Falcon B, Zhang W, Murzin AG, Murshudov G, Garringer HJ, Vidal R, Crowther RA, Ghetti B, Scheres SHW, Goedert M (2018). Structures of filaments from Pick’s disease reveal a novel tau protein fold. Nature.

[164] Falcon B, Zivanov J, Zhang W, Murzin AG, Garringer HJ, Vidal R, Crowther RA, Newell KL, Ghetti B, Goedert M, Scheres SHW (2019). Novel tau filament fold in chronic traumatic encephalopathy encloses hydrophobic molecules. Nature.

[165] Zhang W, Tarutani A, Newell KL, Murzin AG, Matsubara T, Falcon B, Vidal R, Garringer HJ, Shi Y, Ikeuchi T (2020). Novel tau filament fold in corticobasal degeneration. Nature.

[166] Arakhamia T, Lee CE, Carlomagno Y, Duong DM, Kundinger SR, Wang K, Williams D, DeTure M, Dickson DW, Cook CN (2020). Posttranslational Modifications Mediate the Structural Diversity of Tauopathy Strains. Cell.

[167] Shi Y, Zhang W, Yang Y, Murzin A, Falcon B, Kotecha A, van Beers M, Tarutani A, Kametani F, Garringer HJ, et al. Structure-based Classification of Tauopathies. bioRxiv 2021. 10.1038/s41586-021-03911-7 PMC7611841 34588692

[168] Tagliafierro L, Bonawitz K, Glenn OC, Chiba-Falek O (2016). Gene Expression Analysis of Neurons and Astrocytes Isolated by Laser Capture Microdissection from Frozen Human Brain Tissues. Front Mol Neurosci.

[169] Poorkaj P, Bird TD, Wijsman E, Nemens E, Garruto RM, Anderson L, Andreadis A, Wiederholt WC, Raskind M, Schellenberg GD (1998). Tau is a candidate gene for chromosome 17 frontotemporal dementia. Ann Neurol.

[170] Hutton M, Lendon CL, Rizzu P, Baker M, Froelich S, Houlden H, Pickering-Brown S, Chakraverty S, Isaacs A, Grover A (1998). Association of missense and 5’-splice-site mutations in tau with the inherited dementia FTDP-17. Nature.

[171] Clark LN, Poorkaj P, Wszolek Z, Geschwind DH, Nasreddine ZS, Miller B, Li D, Payami H, Awert F, Markopoulou K (1998). Pathogenic implications of mutations in the tau gene in pallido-ponto-nigral degeneration and related neurodegenerative disorders linked to chromosome 17. Proc Natl Acad Sci USA.

[172] Dujardin S, Colin M, Buee L (2015). Invited review: Animal models of tauopathies and their implications for research/translation into the clinic. Neuropathol Appl Neurobiol.

[173] Brion JP, Tremp G, Octave JN (1999). Transgenic expression of the shortest human tau affects its compartmentalization and its phosphorylation as in the pretangle stage of Alzheimer’s disease. Am J Pathol.

[174] Gotz J, Tolnay M, Barmettler R, Chen F, Probst A, Nitsch RM (2001). Oligodendroglial tau filament formation in transgenic mice expressing G272V tau. Eur J Neurosci.

[175] Lewis J, McGowan E, Rockwood J, Melrose H, Nacharaju P, Van Slegtenhorst M, Gwinn-Hardy K, Paul Murphy M, Baker M, Yu X (2000). Neurofibrillary tangles, amyotrophy and progressive motor disturbance in mice expressing mutant (P301L) tau protein. Nat Genet.

[176] Lin WL, Lewis J, Yen SH, Hutton M, Dickson DW (2003). Filamentous tau in oligodendrocytes and astrocytes of transgenic mice expressing the human tau isoform with the P301L mutation. Am J Pathol.

[177] Santacruz K, Lewis J, Spires T, Paulson J, Kotilinek L, Ingelsson M, Guimaraes A, DeTure M, Ramsden M, McGowan E (2005). Tau suppression in a neurodegenerative mouse model improves memory function. Science.

[178] Ren Y, Lin WL, Sanchez L, Ceballos C, Polydoro M, Spires-Jones TL, Hyman BT, Dickson DW, Sahara N (2014). Endogenous tau aggregates in oligodendrocytes of rTg4510 mice induced by human P301L tau. J Alzheimers Dis.

[179] Higuchi M, Ishihara T, Zhang B, Hong M, Andreadis A, Trojanowski J, Lee VM (2002). Transgenic mouse model of tauopathies with glial pathology and nervous system degeneration. Neuron.

[180] Dawson HN, Cantillana V, Chen L, Vitek MP (2007). The tau N279K exon 10 splicing mutation recapitulates frontotemporal dementia and parkinsonism linked to chromosome 17 tauopathy in a mouse model. J Neurosci.

[181] Forman MS, Lal D, Zhang B, Dabir DV, Swanson E, Lee VM, Trojanowski JQ (2005). Transgenic mouse model of tau pathology in astrocytes leading to nervous system degeneration. J Neurosci.

[182] Higuchi M, Zhang B, Forman MS, Yoshiyama Y, Trojanowski JQ, Lee VM (2005). Axonal degeneration induced by targeted expression of mutant human tau in oligodendrocytes of transgenic mice that model glial tauopathies. J Neurosci.

[183] Kouri N, Ross OA, Dombroski B, Younkin CS, Serie DJ, Soto-Ortolaza A, Baker M, Finch NCA, Yoon H, Kim J (2015). Genome-wide association study of corticobasal degeneration identifies risk variants shared with progressive supranuclear palsy. Nat Commun.

[184] Ferrer I, Garcia MA, Gonzalez IL, Lucena DD, Villalonga AR, Tech MC, Llorens F, Garcia-Esparcia P, Martinez-Maldonado A, Mendez MF (2018). Aging-related tau astrogliopathy (ARTAG): not only tau phosphorylation in astrocytes. Brain Pathol.

[185] Narasimhan S, Changolkar L, Riddle DM, Kats A, Stieber A, Weitzman SA, Zhang B, Li Z, Roberson ED, Trojanowski JQ, Lee VMY. Human tau pathology transmits glial tau aggregates in the absence of neuronal tau. J Exp Med. 2020;217. 10.1084/jem.20190783 PMC7041709 31826239

[186] He Z, McBride JD, Xu H, Changolkar L, Kim SJ, Zhang B, Narasimhan S, Gibbons GS, Guo JL, Kozak M (2020). Transmission of tauopathy strains is independent of their isoform composition. Nat Commun.

[187] Dickson DW, Ahmed Z, Algom AA, Tsuboi Y, Josephs KA (2010). Neuropathology of variants of progressive supranuclear palsy. Curr Opin Neurol.

[188] Fu H, Hardy J, Duff KE (2018). Selective vulnerability in neurodegenerative diseases. Nat Neurosci.

[189] Roussarie JP, Yao V, Rodriguez-Rodriguez P, Oughtred R, Rust J, Plautz Z, Kasturia S, Albornoz C, Wang W, Schmidt EF (2020). Selective Neuronal Vulnerability in Alzheimer’s Disease: A Network-Based Analysis. Neuron.

[190] Roux KJ, Kim DI, Burke B, May DG. BioID: A Screen for Protein-Protein Interactions. Curr Protoc Protein Sci 2018;91:19 23 11–19 23 15. 10.1002/cpps.51 PMC6028010 29516480

[191] Ping L, Duong DM, Yin L, Gearing M, Lah JJ, Levey AI, Seyfried NT (2018). Global quantitative analysis of the human brain proteome in Alzheimer’s and Parkinson’s Disease. Sci Data.

[192] Johnson ECB, Dammer EB, Duong DM, Yin L, Thambisetty M, Troncoso JC, Lah JJ, Levey AI, Seyfried NT (2018). Deep proteomic network analysis of Alzheimer’s disease brain reveals alterations in RNA binding proteins and RNA splicing associated with disease. Mol Neurodegener.

[193] Congdon EE, Sigurdsson EM (2018). Tau-targeting therapies for Alzheimer disease. Nat Rev Neurol.

[194] Jadhav S, Avila J, Scholl M, Kovacs GG, Kovari E, Skrabana R, Evans LD, Kontsekova E, Malawska B, de Silva R (2019). A walk through tau therapeutic strategies. Acta Neuropathol Commun.

[195] Long JM, Holtzman DM (2019). Alzheimer Disease: An Update on Pathobiology and Treatment Strategies. Cell.

[196] Spillantini MG, Goedert M (2000). The alpha-synucleinopathies: Parkinson’s disease, dementia with Lewy bodies, and multiple system atrophy. Ann N Y Acad Sci.

[197] Lee EB, Porta S, Michael Baer G, Xu Y, Suh E, Kwong LK, Elman L, Grossman M, Lee VM, Irwin DJ (2017). Expansion of the classification of FTLD-TDP: distinct pathology associated with rapidly progressive frontotemporal degeneration. Acta Neuropathol.

[198] Mackenzie IR, Neumann M (2017). Reappraisal of TDP-43 pathology in FTLD-U subtypes. Acta Neuropathol.

[199] Peng C, Gathagan RJ, Covell DJ, Medellin C, Stieber A, Robinson JL, Zhang B, Pitkin RM, Olufemi MF, Luk KC (2018). Cellular milieu imparts distinct pathological alpha-synuclein strains in alpha-synucleinopathies. Nature.

[200] Laferriere F, Maniecka Z, Perez-Berlanga M, Hruska-Plochan M, Gilhespy L, Hock EM, Wagner U, Afroz T, Boersema PJ, Barmettler G (2019). TDP-43 extracted from frontotemporal lobar degeneration subject brains displays distinct aggregate assemblies and neurotoxic effects reflecting disease progression rates. Nat Neurosci.

